# Metastable Structure for Ultra‐Sustainable, High Capacity and Kinetics‐Enhanced Magnesium‐Ion Battery

**DOI:** 10.1002/advs.202522416

**Published:** 2026-01-04

**Authors:** Rongrui Deng, Yumei Wang, Zhongting Wang, Xingyang Wang, Chaoneng Dai, Lingxiao Luo, Yue Guo, Jiaqi Peng, Zhenhang Huang, Shuangshuang Tan, Hongyi Li, Fusheng Pan, John Wang

**Affiliations:** ^1^ National University of Singapore (Chongqing) Research Institute Chongqing China; ^2^ National Engineering Research Center For Magnesium Alloys College of Materials Science and Engineering Chongqing University Chongqing China; ^3^ Department of Materials Science and Engineering National University of Singapore Singapore Singapore; ^4^ School of Materials and Energy Southwest University Chongqing China; ^5^ College of Materials Xiamen University Xiamen China

**Keywords:** magnesium‐ion batteries, metastable phase evolution, Mg^2+^‐storage capability

## Abstract

Cost‐effective magnesium ion batteries (MIBs) offer a promising new pathway for next‐generation large‐scale energy storage, and yet its development is largely hindered by the severe polarization, limited rate capability, and poor cycling stability, where the challenges are largely rooted in the sluggish kinetics of Mg^2+^ storage. Here, we report a metastable phase evolution strategy that enables high‐performance Mg^2+^ storage by leveraging Ti‐modulated VS_4_ (T‐VS_4_), demonstrating a structurally soft and yet dynamically adaptive lattice, which are among the preconditions for the metastable phase formation. Metastable Mg_x_T‐VS_4_ is formed during the initial Mg^2+^ intercalation, significantly facilitating the subsequent Mg^2+^ migration, favoring multi‐electron redox reaction, and enhancing the charge transfer kinetics. Impressively, the T‐VS_4_ cathode demonstrates exceptional Mg^2+^ storage performance with a high specific capacity (205.4 mAh g^−1^ at 50 mA g^−1^), excellent rate capability (up to 1000 mA g^−1^), and long‐term cycling stability (over 3000 cycles). This work exemplifies a new metastable phase engineering approach as the design paradigm for breaking kinetic limitations in MIBs, offering a novel avenue toward next‐generation energy storage systems.

## Graphical abstract

A metastable‐phase evolution strategy is proposed for magnesium‐ion batteries, enabling the in situ formation of a metastable Mg_x_T‐VS_4_ cathode during cycling. The metastable state reshapes Mg^2+^ storage kinetics and enhances structural stability, offering a new design paradigm for multivalent ion battery cathodes.

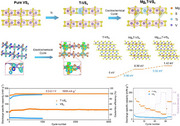

## 1 Introduction

The rapid on‐going of economic growth and industrialization have driven an ever‐rising demand for efficient and stable energy storage systems. Although lithium‐ion batteries have achieved the large commercial maturity, its reliance on geologically scarce lithium resources and escalating production costs limits their largescale applications [1, 2, 3]. Magnesium‐ion batteries (MIBs), leveraging earth‐abundant magnesium and its divalent charge, offer intrinsic safety and high volumetric capacity, positioning them as a compelling alternative to lithium‐ion battery systems [4, 5, 6]. However, the strong electrostatic interactions inherent to divalent ions pose significant challenges, limiting ion diffusion kinetics and slowing intercalation reactions [7, 8, 9]. These interactions impose high migration barriers and trigger irreversible structural distortions during cycling, resulting in poor rate capability and limited lifespan [10, 11]. Conventional cathode design for MIBs, guided by thermodynamic stability, prioritizes the rigid, phase‐persistent structure. However, such materials often lack the dynamic flexibility required to accommodate divalent ion transport, presenting a fundamental contradiction between the structural stability and functional performance [12].

This contradiction calls for a rethinking of design principles beyond the thermodynamic ground state. Metastable phases, non‐equilibrium structures residing in local energy minima, offer a compelling alternative [13, 14, 15]. Unlike their equilibrium counterparts, metastable structural configurations can exhibit softer lattices, richer electronic structures, and greater redox diversity, all of which are favorable for fast ion transport and reversible multielectron storage [16, 17]. Nevertheless, leveraging metastability in MIB systems has remained largely conceptual [18, 19]. The practical realization of metastable phases faces intrinsic difficulties: they are often inaccessible by conventional synthesis and inherently prone to relaxation into more stable configurations [20, 21]. Stabilizing these phases, where structural strain and redox stress are pronounced, is particularly challenging [22, 23].

To overcome this barrier, we adopt a fundamentally different strategy: instead of attempting to pre‐synthesize metastable materials, we harness the electrochemical reactions during cycling as a means of in situ generating and stabilizing metastable states. Based upon this principle, we design a host lattice of Ti‐modified VS_4_ to induce a metastable phase transition during Mg^2+^ insertion. VS_4_ is a redox‐flexible sulfide with mixed cationic‐anionic activity, and its 1D chain structure provides a platform amenable to dynamic reconfiguration [24, 25]. Density functional theory (DFT) calculations suggest that the incorporation of Ti into VS_4_ leads to lattice softening and sulfur‐vacancy generation, which collectively narrow the electronic bandgap and increase the density of states near the Fermi level. These changes could enhance the electronic conductivity and Mg^2+^ affinity of the host, and more critically, create a structurally and electronically favorable precursor state that facilitates the subsequent formation of a metastable phase (Mg_x_T‐VS_4_). Benefiting from this metastable structure, the resultant battery demonstrates an excellent discharge specific capacity of 205.4 mAh g^−1^ at the current density of 50 mA g^−1^ (∼8 times than that of pure VS_4_). Under the high current density of 1 A g^−1^, the battery could work stably over 3000 cycles. Beyond its immediate performance, this work demonstrates a new design paradigm in which metastability is not merely tolerated but deliberately accessed and harnessed through controlled electrochemical dynamics. By decoupling functional performance from equilibrium constraints, we provide a pathway toward metastability‐engineered cathodes for high‐performance MIB systems and beyond.

## 2 Results and Discussion

### 2.1 Structural Evolution of Precursor T‐VS_4_

**FIGURE 1:**
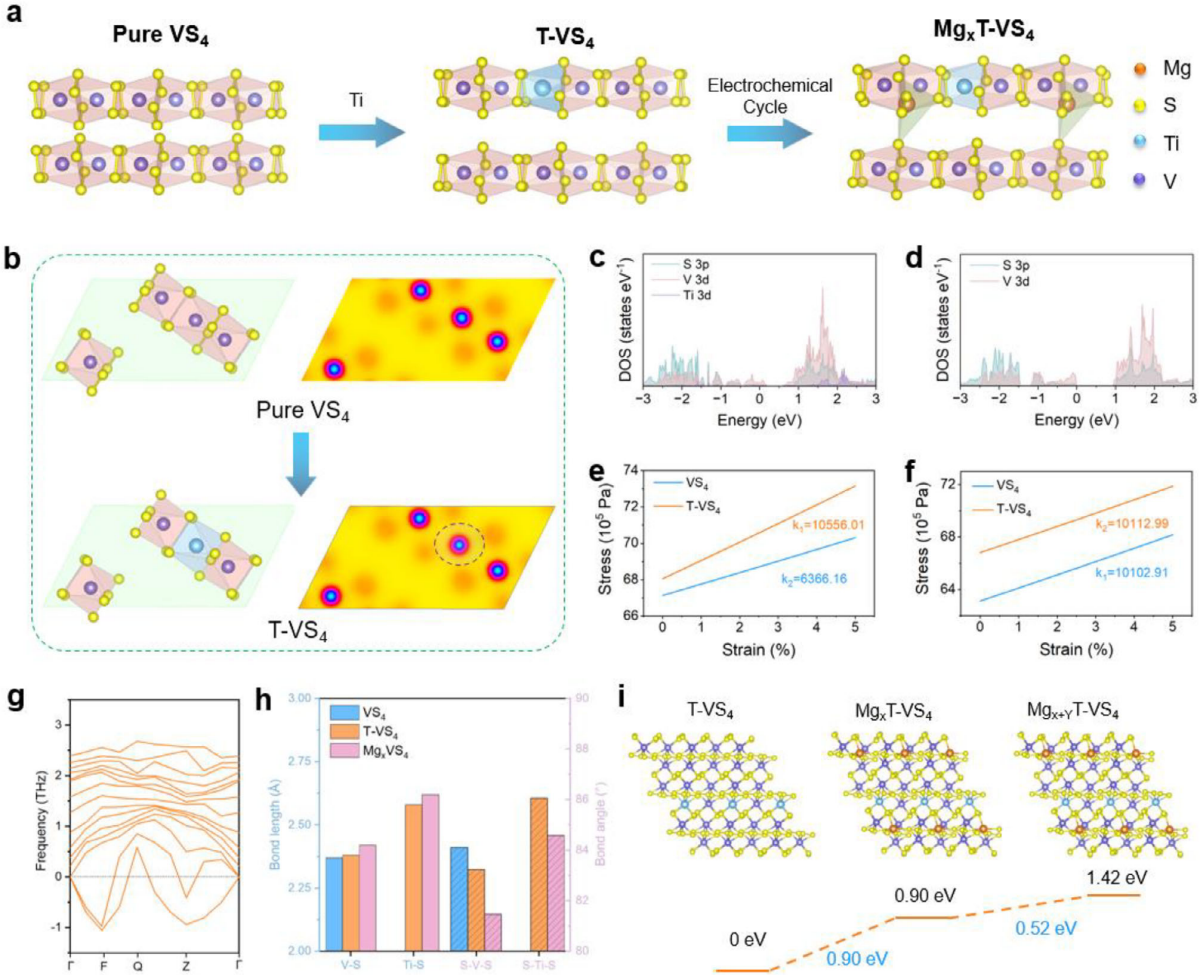
Material design principles and theoretical simulation. (a) Schematic illustration of material design. (b) ELF of VS_4_ and T‐VS_4_. Calculated density of states of (c) T‐VS_4_ and (d) VS_4_. Calculated Young's modulus along the (e) *(010)* and (f) *(100)* planes of T‐VS_4_ and VS_4_. (g) The phonon spectrum of Mg_x_T‐VS_4_. (h) The changes in bond length and bond angle of the VS_4_, T‐VS_4_, and Mg_x_T‐VS_4_. (i) The energy required for forming metastable structures (Mg_x_T‐VS_4_) and continuing magnesium intercalation (Mg_x+y_T‐VS_4_).

We employ Ti‐modified VS_4_ (T‐VS_4_) as the structural template to guide the in situ formation of a metastable Mg_x_T‐VS_4_ phase during electrochemical cycling. The 1D linear chain structure of VS_4_ provides inherent structural anisotropy and redox flexibility, while the incorporation of Ti ions (ionic radius: 0.61 Å, larger than that of V at 0.58 Å) subtly tunes the local coordination environment, introducing sulfur vacancies and expanding interlayer spacing, which perturbs the local bonding symmetry as well as enhances electronic delocalization, thereby weakening the rigid ground‐state configuration [26, 27, 28]. These structural perturbations induce electron delocalization and lattice softening, effectively destabilizing the thermodynamic ground state and creating a thermodynamically constrained but kinetically accessible pathway for the evolution and stabilization of the metastable Mg_x_T‐VS_4_ phase during Mg^2+^ insertion (Figure 1a). Notably, Ti substitution does more than generate isolated sulfur vacancies. By simultaneously modifying the local coordination and tuning the chain environment, it reshapes the energy landscape in a coordinated manner that lowers the kinetic barrier for the transformation into the metastable phase. Thus, the metastable Mg_x_T‐VS_4_ phase originates from a Ti‐regulated adjustment of local bonding and chain configuration, rather than from a simple vacancy effect.

Insights from the electron localization function (ELF) in Figure 1b reveal a pronounced transformation in bonding character. Upon Ti substitution, the ELF contour becomes significantly delocalized around Ti─S bonds, suggesting a reduction in electron density sharing and a weakening of local bond strength. This delocalization is attributed to the lower electronegativity and distinct orbital hybridization of Ti^4+^ over V^4+^, which disrupts the original bonding symmetry and introduces local lattice inhomogeneity. The resultant softening framework would increase structural compliance and facilitates Mg^2+^ insertion by reducing the diffusion energy barrier, which provides the electronic and structural driving force for the in situ evolution of a metastable Mg_x_T‐VS_4_ phase. To elucidate the electronic and mechanical origins of metastable phase formation, we conducted a series of first‐principles calculations. As demonstrated in the DOS calculation (Figure 1c,d; Figures S1–S7), Ti introduces shallow acceptor states near the Fermi level, effectively narrowing the bandgap and enhancing the density of available electronic states. This modulation promotes electron hopping and charge delocalization, which are critical for sustaining rapid redox kinetics under nonequilibrium cycling conditions. Moreover, the upward shift in S p and Ti d states in T‐VS_4_ suggests an increase in anionic redox activity, further supporting multi‐electron transfer. To investigate the mechanical effect, we performed uniaxial strain simulations based on classical molecular dynamics by applying up to 5% strain along *(010)* and *(100*) planes. As presented in Figure 1e,f, T‐VS_4_ consistently exhibits a higher Young's modulus compared to the pristine structure across all crystallographic planes, indicating enhanced lattice stiffness and a mechanically reinforced framework. This mechanical strengthening arises from the substitution of smaller‐radius V^4+^ with larger and more electropositive Ti^4+^, which reinforces the ─S bonding network and reduces the structural compliance under stress. Additionally, the enhanced stiffness could mitigate irreversible structural distortions during Mg^2+^ insertion/extraction, suppresses the local collapse phenomena, and promotes long‐range structural coherence, which are among the keys for the formation and long‐term cycling stability of the metastable phases.

To further ascertain the metastable nature of Mg_x_T‐VS_4_ and its implications for Mg^2+^ storage, we performed detailed theoretical evaluations. Phonon dispersion calculations of Mg_x_T‐VS_4_ in Figure 1g reveal the presence of imaginary frequencies, confirming that the material is thermodynamically unstable and thus resides in a metastable state. Structural analysis further reveals that Ti incorporation and subsequent metastable evolution collectively elongate V─S and Ti─S bond lengths while reducing S─V─S and S─Ti─S bond angles (Table S1; Figure 1h). This bond relaxation enlarges local polyhedral volumes and weakens angular constraints, creating a softer and more deformable framework. This structural compliance lowers the steric hindrance and electrostatic penalty typically associated with Mg^2+^ intercalation, thereby facilitating ion transport and insertion. The corresponding energy profile illustrated in Figure 1i shows that while the in‐ situ formation of the metastable Mg_x_T‐VS_4_ requires an initial barrier of 0.9 eV, subsequent Mg^2+^ incorporation into this framework proceeds with a significantly reduced energy demand of 0.52 eV. This energetic descent highlights that once established, the metastable phase can stabilize under cycling and also offers a kinetically favorable host for further Mg^2+^ storage.

**FIGURE 2:**
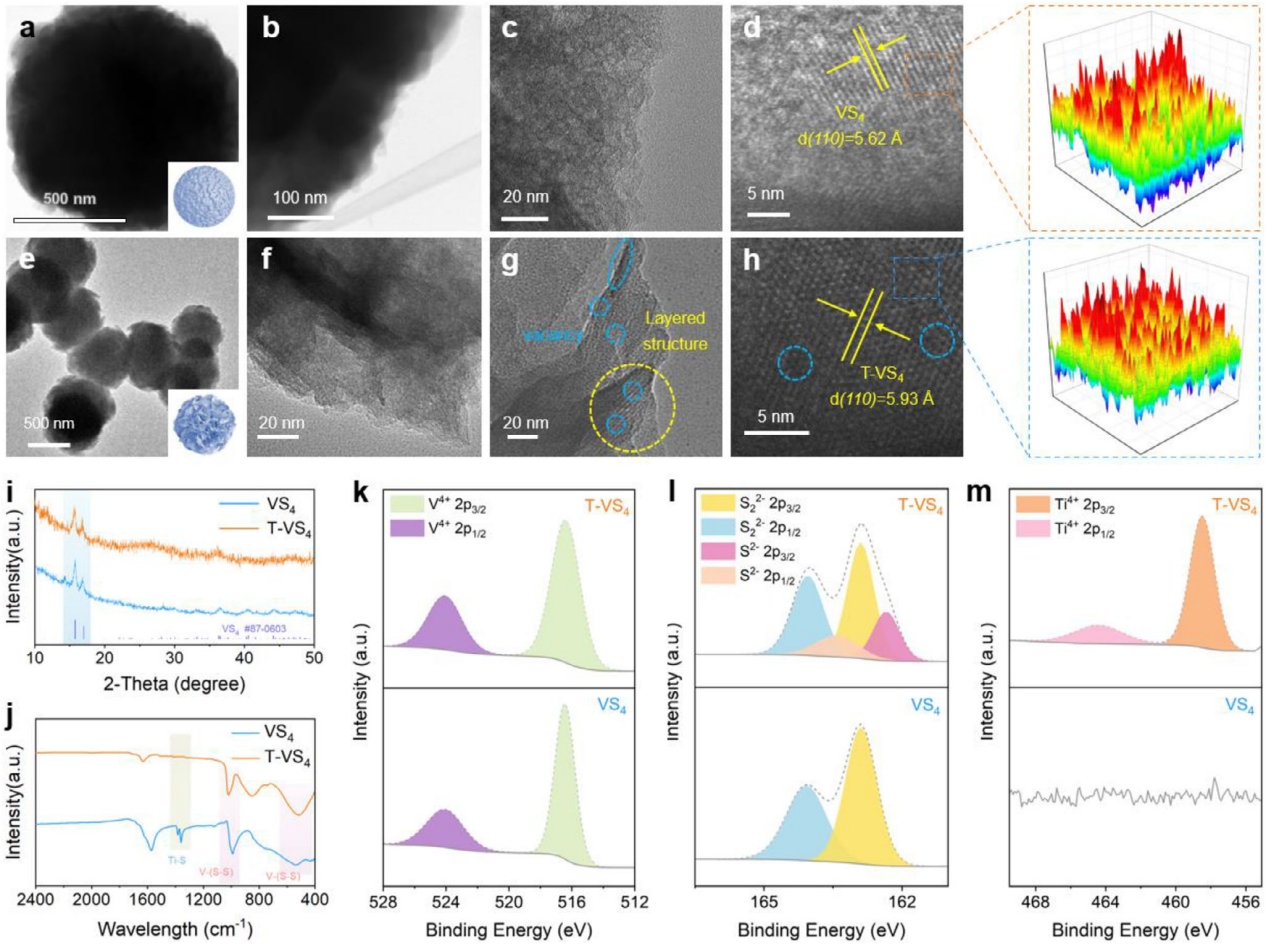
Morphology, microstructural, and chemical composition characterization. (a–c) STEM image, (d) HR‐TEM image of VS_4_. (e) STEM image, (f–h) HR‐TEM image of T‐VS_4_. (i) XRD results, (j) FT‐IR spectra of the T‐VS_4_ and VS_4_. High‐resolution core level of (k) V 2p, (l) S 2p, and (m) Ti 2p of the T‐VS_4_ and VS_4_.

In Figure 2a, VS_4_ is shown with a solid flower‐like microsphere, composed of stacked nanosheets. High‐magnification images in Figure 2b,c further reveal a dense arrangement at the edges of the microspheres. The lattice spacing of *(110)* is 5.62 Å in the monoclinic VS_4_ (Figure 2d), underscoring the well‐defined crystal structure at the atomic level. By contrast, as shown in Figure 2e, hollow microspheres are observed in T‐VS_4_. These hollow microspheres could effectively facilitate the electrolyte infiltration and increase the contact area between the electrolyte and electrode, thereby helping to enhanced electrochemical performance. The edge region of T‐VS_4_ described in Figure 2f consists of intersecting ultrathin nanosheets. A significant number of defects could be observed in the nanosheets of T‐VS_4_. Besides, a distinct discontinuous lattice, marked by blue circles in Figure 2g, further indicates the presence of abundant sulfur vacancies. In Figure 2h, the lattice spacing in T‐VS_4_ is 5.93 Å, larger than that of VS_4_, indicating the Ti has expanded the interlayer spacing. HR‐TEM images of VS_4_ and T‐VS_4_ were Fourier‐transformed, as shown in right, revealing a homogeneous distribution of S and V atoms, exhibiting a well‐defined crystal structure. In Figures S8 and S9, homogeneous distribution of V and S in VS_4_, and V, S, and Ti in T‐VS_4_ are clearly observed. Through the morphological analyses, Ti could transform solid microspheres into hollow ones, expand the interlayer spacing, and create sulfur vacancies, all of which can contribute to the formation of a metastable structure during the electrochemical process.

To thoroughly investigate the structure evolution of T‐VS_4_, we conducted X‐ray diffraction (XRD) characterization on VS_4_ and T‐VS_4_. In Figure 2i, the diffraction peaks of both T‐VS_4_ and pure VS_4_ are consistent with the standard PDF of VS_4_ (JCPDS No. 87–0603), demonstrating a typical monoclinic structure. As shown in Figure S10 in T‐VS_4_, the *(110)* plane shifts to lower 2θ angle, indicating that the introduction of Ti increases the interlayer spacing of VS_4_, being consistent with the HR‐TEM results. As analyzed by the Fourier Transform Infrared spectroscopy (FTIR) in Figure 2j, the characteristic peaks of the V─(S─S) bond (∼550 cm^−1^ and ∼980 cm^−1^) present a slight blueshift in T‐VS_4_. Moreover, an additional Ti─S bond is detected at 1380 cm^−1^ in T‐VS_4_, confirming Ti has been successfully bonded to the VS_4_ lattice.

The chemical environments of VS_4_ and T‐VS_4_ have been further investigated via the X‐ray photoelectron spectroscopy (XPS), and Ti could be only observed in T‐VS_4_ (Figure S11). Figure 2k displays the high‐resolution XPS spectra of V 2p, showing two primary peaks at 516.4 and 524.1 eV corresponding to V^4+^ 2p_3/2_ and 2p_1/2_, in both T‐VS_4_ and pure VS_4_, which indicates Ti does not alter the valence state of vanadium. However, notable differences in S are observed between T‐VS_4_ and VS_4_ (Figure 2l). In VS_4_, two distinct peaks of S_2_^2−^ 2p_3/2_ at 162.9 eV and S_2_^2−^ 2p_1/2_ at 164.1 eV are observed, corresponding to the characteristic feature of V─(S─S). Meanwhile, two additional peaks at 162.3 and 163.4 eV are observed in T‐VS_4_, which should be attributed to the S^2−^ 2p_3/2_ and S^2−^ 2p_1/2_, indicating the formation of Ti─(S─S) bond. In Figure 2m, the appearance of Ti^4+^ 3d_3/2_ (458.5 eV) and Ti^4+^ 3d_1/2_ (464.4 eV) peaks further confirms that Ti^4+^ has successfully replaced V^4+^ in T‐VS_4_ to form Ti‐S bonds. Specifically, in the lattice of VS_4_, each V atom bonds with four S atoms. Upon Ti introducing, Ti would replace some of V and bond with two S, forming the Ti─(S─S) bond. This substitution disrupts the original V─S bond, resulting in the formation of isolated sulfur species that may eventually escape, leading to the creation of a substantial number of sulfur vacancies. As shown in Figure S12, a resonance signal with a *g*‐value of 1.9763 has been detected via the electron paramagnetic resonance (EPR) spectroscopy, indicating that sulfur vacancies have been successfully introduced into T‐VS_4_.

### 2.2 Metastable Mg_x_T‐VS_4_ Formation and Enhanced Mg^2+^ Storage Capability

**FIGURE 3:**
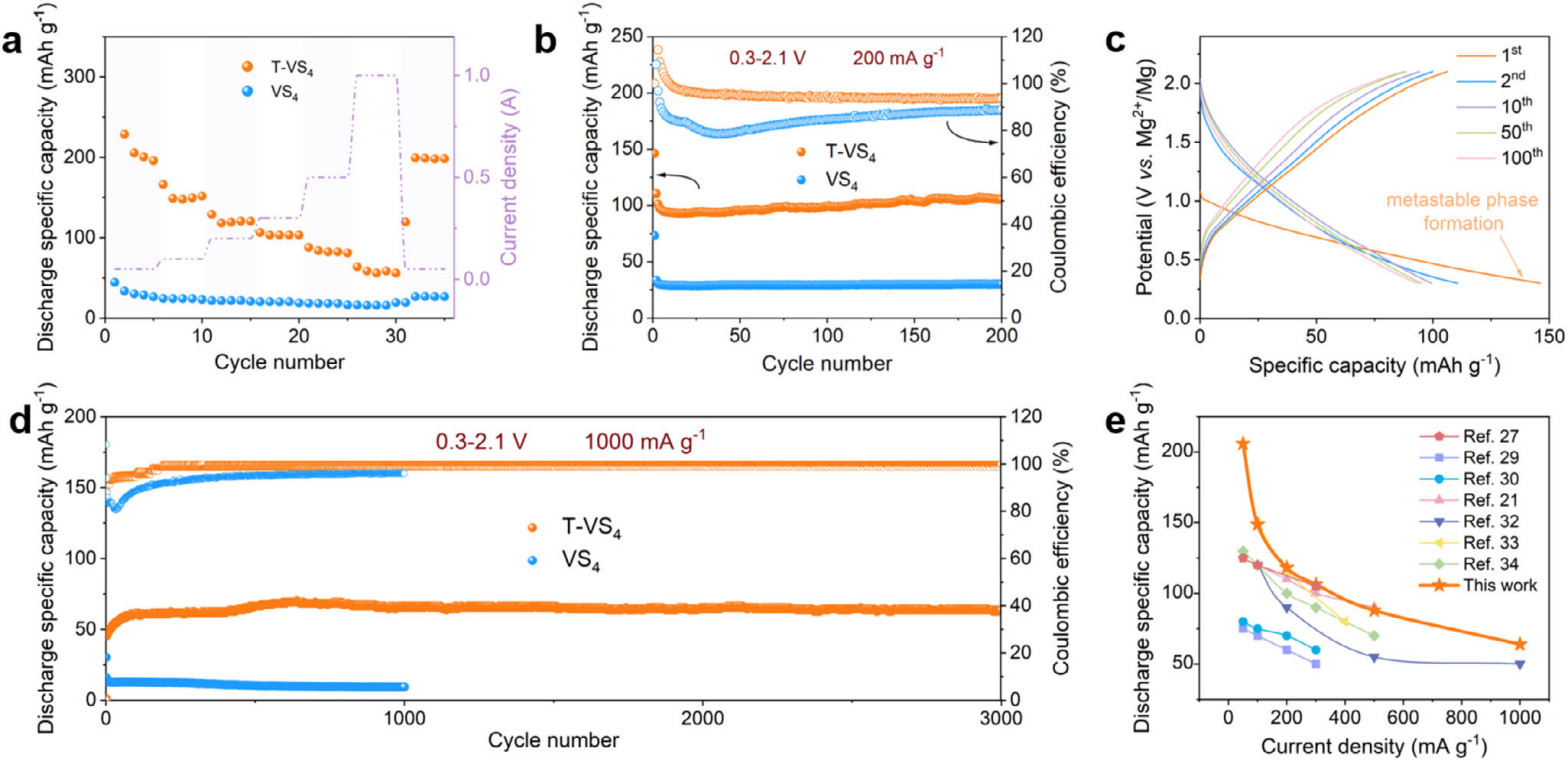
Electrochemical performance of the T‐VS_4_ and VS_4_ cathodes. (a) Rate capability, (b) Cycling performance of the T‐VS_4_ and VS_4_ cathodes at 200 mA g^−1^. (c) GCD curves of T‐VS_4_ cathode under different cycles at 200 mA g^−1^. (d) Long‐term cycling performance of the T‐VS_4_ cathode at 1000 mA g^−1^. (e) The electrochemical performance comparison of T‐VS_4_ with other VS_4_‐based cathodes.

In this study, we investigate the role of a metastable Mg_x_T‐VS_4_ phase formed in T‐VS_4_ upon initial Mg^2+^ insertion, which significantly improves the electrochemical performance of MIBs. All electrochemical tests were performed using the APC ((MgPhCl)_2_‐AlCl_3_ in THF) electrolyte. Unlike the typical stable structures that form in conventional materials, the metastable T‐VS_4_ allows the formation of another metastable phase that is stabilized by Ti‐modified and Mg^2+^ intercalation. This phase is structurally distinct from the original VS_4_, yet it retains key features of the original structure, ensuring dynamic adaptability and continuous structural reorganization throughout cycling. This unique metastable phase facilitates the efficient Mg^2+^ diffusion, reduces migration barriers, and enables multi‐electron redox reactions involving both vanadium and sulfur species. To confirm the formation and stability of this metastable phase, cyclic voltammetry (CV) was conducted. As shown in Figures S13 and S14, an obvious irreversible reduction peak at ∼0.6 V is observed in the initial CV curve in both VS_4_ and T‐VS_4_, implying a Mg‐rich phase is formed during the initial Mg^2+^ insertion. However, only T‐VS_4_ demonstrates stable Mg^2+^ insertion and extraction in subsequent cycles. The initial irreversible reduction peak corresponds to the formation of the metastable Mg_x_T‐VS_4_ phase, during which a portion of Mg^2+^ is trapped to stabilize the structure. Once this metastable phase is established, subsequent Mg^2+^ insertion and extraction proceed more reversibly, mitigating further capacity loss. This stable electrochemical behavior is attributed to the metastable Mg_x_T‐VS_4_ phase, which is dynamically soft and structurally adaptable, unlike the rigid structure of VS_4_ where the Mg^2+^ insertion is essentially “locked” into place. It is structurally distinct and yet topologically related to the parent VS_4_ framework, preserving its fundamental redox‐active motifs while exhibiting expanded interlayer spacing, increased defect density, and locally disordered coordination environments, introducing energetically favorable pathways for Mg^2+^ (de)intercalation. In Figure 3a, the T‐VS_4_ demonstrates superior rate capability over pristine VS_4_. Reversible discharge capacities of 205.4, 149.7, 118.2, 106.4, 87.9, and 64.0 mAh g^−1^ have been achieved in T‐VS_4_, at current densities of 50, 100, 200, 300, 500, and 1000 mA g^−1^, respectively. Upon returning the current density to 50 mA g^−1^, the capacity recovers to 199.1 mAh g^−1^. T‐VS_4_ demonstrates a highly reversible Mg^2+^ insertion/extraction, together with excellent tolerance to current variations. On the other hand, the VS_4_ exhibits limited Mg^2+^ storage capacity and slower kinetics. Figure 3b,c illustrates the cycling performance of T‐VS_4_ at the current density of 200 mA g^−1^, in comparison with VS_4_. The reversible discharge specific capacity of T‐VS_4_ stabilizes at approximately 100 mAh g^−1^, with minimal attenuation over 200 cycles. In contrast, the pristine VS_4_ could only deliver a capacity of 25 mAh g^−1^. As shown in Figure 3d, even at the high current density of 1000 mA g^−1^, the T‐VS_4_ could still deliver an ultra‐stable capacity of 63.5 mA g^−1^ over 3000 cycles, with a coulombic efficiency consistently exceeding 99%. Besides, a gradual capacity increase is observed during the initial cycles at high current densities. This activation behavior arises from the progressive softening of the host framework and the concurrent optimization of the electrode–electrolyte interface, both of which reduce local transport barriers and render additional reaction sites electrochemically accessible. These structural and interfacial adjustments collectively enhance the reversible capacity before the system reaches a steady state. Compared with other reported candidates, superior electrochemical performance has been achieved in our T‐VS_4_ electrode (Figure 3e) [27, 29, 30, 31, 32, 33, 34]. Moreover, we provide a detailed comparison with previously reported VS_4_‐based cathodes for magnesium‐ion batteries in Table S1. These results clearly demonstrate that the metastable Mg_x_T‐VS_4_ phase plays a crucial role in enhancing the electrochemical performance and stability of T‐VS_4_, setting it apart from conventional VS_4_.

**FIGURE 4:**
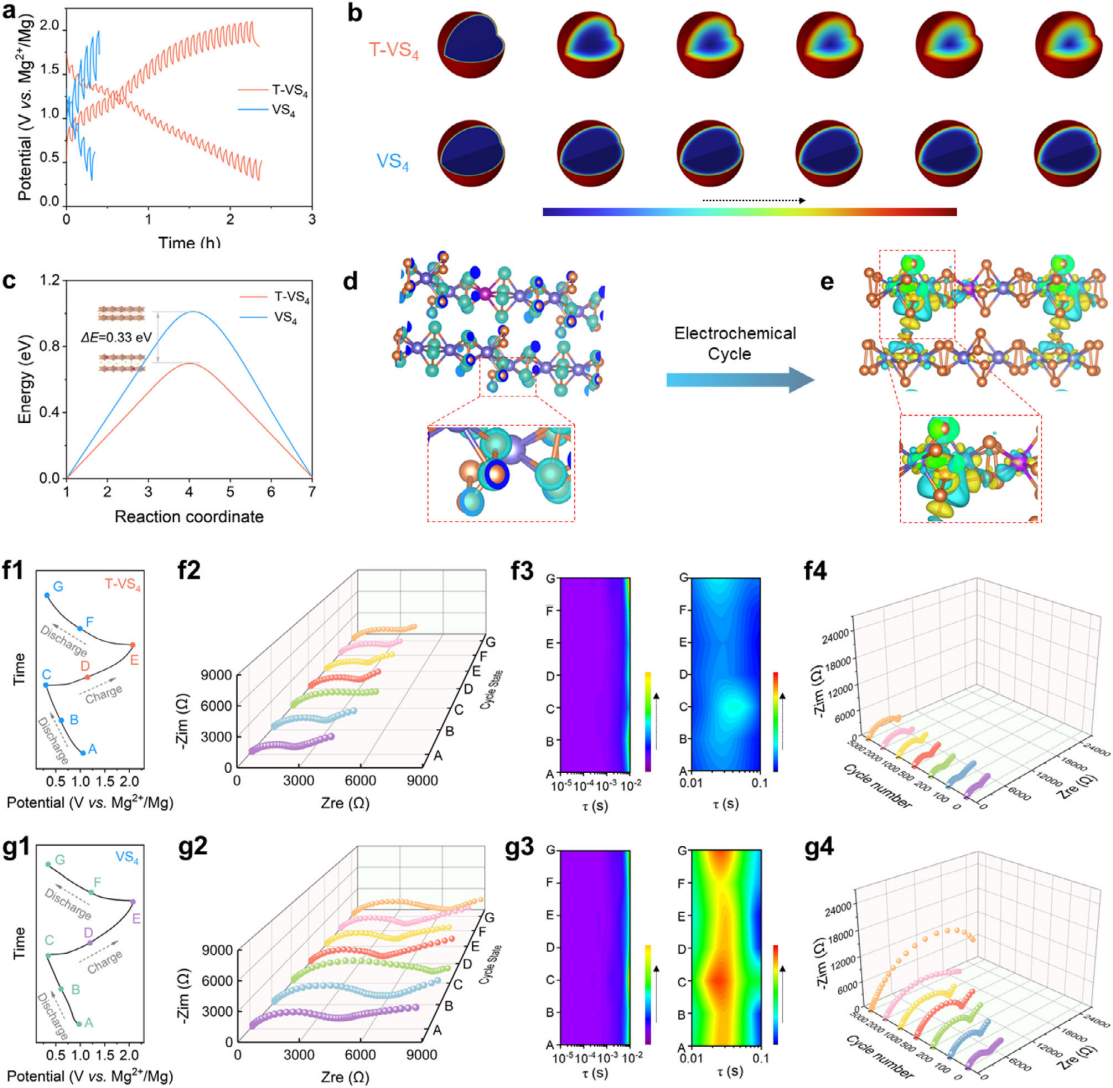
Diffusion kinetics and charge transfer mechanism. (a) GITT profiles, (b) Schematic diagram of the diffusion rate of magnesium ions in T‐VS_4_ and VS_4_. (c) Diffusion energy barrier profiles of Mg^2+^ transport in T‐VS_4_ and VS_4_. Charge density difference of T‐VS_4_ (d) before and (e) after Mg^2+^ insertion. (f1) Discharge/charge profiles at various states, (f2) corresponding GEIS results, (f3) DRT transition results, (f4) EIS results before cycle and after various cycles of T‐VS_4_ cathode. (g1) Discharge/charge profiles at various states, (g2) corresponding GEIS results, (g3) DRT transition results, (g4) EIS results before cycle and after various cycles of VS_4_ cathode.

To evaluate the Mg^2+^ diffusion kinetics in VS_4_ and T‐VS_4_, Galvanostatic Intermittent Titration Technique (GITT) experiments were conducted (Figure 4a; Equation S1). As illustrated in Figure S15, the corresponding diffusion coefficient *D(_Mg_^2+^)* of T‐VS_4_ ranges from 10^−8^ to 10^−11^ cm^2^ s^−1^ over a complete discharge/charge cycle, whereas for VS_4_, it falls within the range of 10^−9^ to 10^−12^ cm^2^ s^−1^, suggesting that the metastable phases along with abundant sulfur vacancies and the expanded interlayer spacing in T‐VS_4_ do effectively accelerate the Mg^2+^ diffusion. Mg^2+^ ion diffusion trajectory simulations were conducted based on the GITT‐derived diffusion coefficients. As shown in Figure 4b, T‐VS_4_ exhibits more continuous and isotropic diffusion pathways, whereas the diffusion in pristine VS_4_ appears more restricted. These findings confirm that the metastable Mg_x_T‐VS_4_ framework stabilizes a more open and flexible lattice configuration and thereby enables more accessible ion migration channels. Moreover, the average internal resistance for T‐VS_4_ is 1.42 Ω during discharge and 2.33 Ω during charge, compared to 7.59 Ω in discharge and 11.28 Ω in charge for VS_4_ (Figures S16 and S17), further revealing a much reduced diffusion barrier, consistent with the calculation results in Figure 4c. Furthermore, T‐VS_4_ exhibits a diffusion‐controlled ion storage, contrasting with VS_4_ where pseudo‐surface capacitive behavior dominates (Figures S18–S25), most probably due to the abundant sulfur vacancies and expanded spacings in the metastable phase in T‐VS_4_. Based on DFT calculations, clear charge accumulation has been observed between Mg and the adjacent S in T‐VS_4_, as shown in Figure 4d,e, indicating strong orbital hybridization and enhanced bonding interactions. This charge accumulation facilitates the formation of a metastable, charge‐delocalized phase, which is further evidenced by the redistribution of electronic density around the V─S framework. The redistribution of charge density results in a more flexible and dynamic electronic structure, which effectively lowers the energy barriers for both Mg^2+^ diffusion and charge transfer. This dynamic structure is critical to the improved electrochemical behavior observed in T‐VS_4_.

To further evaluate the charge transfer kinetics in VS_4_/T‐VS_4_, the electrochemical impedance at various discharge and charge states were recorded in both cathodes (Figure 4f1,g1). As shown in Figure 4f2,g2, obviously lower total impedance has been achieved in T‐VS_4_, attributed to its optimized crystalline structure, providing more storage sites and wider pathways for Mg‐ion transport. The impedance of the T‐VS_4_ at the fully charged (“e”) does not completely return to the initial value observed at the beginning, instead, a moderate yet stable deviation is observed, indicating that the electrochemical process leads to a persistent structural transformation rather than the full recovery to the original lattice configuration. This behavior provides compelling evidence that the electrochemical process does not simply revert to the original VS_4_ framework but evolves into a new metastable configuration optimized for Mg^2+^ diffusion and charge transfer. Thus, the difference in impedance before and after a full cycle serves as an electrochemical fingerprint of the metastable phase formation. The Distribution of Relaxation Time (DRT) analysis has also been employed. In DRT analysis, the shorter relaxation time often relates to ion diffusion resistance within the electrolyte, whereas the longer relaxation time corresponds to the interfacial resistances, including the charge transfer and SEI resistances [35, 36]. As shown in Figure 4f3,g3, during discharge, the formation of the Mg_x_T‐VS_4_ along with the Mg stripping at the anode, leading to a significant τ (10^−2^∼10^−1^) change during cycling. Obviously, Compared with VS_4_, T‐VS_4_ presents a much lower impedance at the τ value of 10^−2^∼10^−1^, indicating a faster charge transfer process. Moreover, the electrochemical impedance of VS_4_ and T‐VS_4_ cells have been further recorded after long‐term cycling. In Figure 4f4,g4, the impedance of the VS_4_ cell increases significantly, rising up from ∼6000 Ω in the first cycle to ∼24000 Ω after 5000 cycles, while the impedance in T‐VS_4_ cell maintains at a lower value (∼6000 Ω) over 5000 cycles. Obviously, enhanced charge transfer kinetics and long‐term electrochemical stability have been achieved in T‐VS_4_.

### 2.3 Metastable Mg_x_T‐VS_4_ Formation Mechanism

**FIGURE 5:**
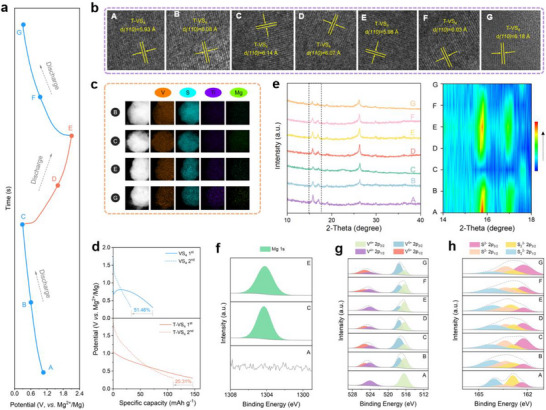
Metastable Mg_x_T‐VS_4_ formation mechanism. (a) The discharge/charge profiles of T‐VS_4_ cathodes at different discharge and charge states. (b) Ex situ TEM images, (c) TEM‐EDS mappings of T‐VS_4_ cathodes at different stage, (d) capacity loss at C and G states. and (e) ex situ XRD patterns and corresponding enlarged *(110)* and *(020)* peaks, and. High‐resolution ex situ XPS spectra of (f) Mg 1s, (g) V 2p, (h) S 2p.

To thoroughly understand the metastable Mg_x_T‐VS_4_ formation process in T‐VS_4_, STEM, XRD, and XPS analysis were systematically conducted on T‐VS_4_, at typical stages during discharging/charging (Figure 5a), to investigate its structure evolution, and phase/composition changes. Figure 5b tracks the lattice spacing change in T‐VS_4_. Initially, T‐VS_4_ demonstrates a monoclinic prismatic structure with the lattice spacing of *(110)* plane at 5.93 Å (stage A). During magnesiation, the spacing increases progressively to 6.08 Å at stage B, eventually reaching 6.14 Å at stage C. In the following charge process (from state C to E), it gradually decreases to 5.98 Å at the fully charged state E. Notably, the spacing at E remains slightly larger than that at the original state A, suggesting the emergence of a distinct phase with partial Mg^2+^ retention. Such structural evolution and partial Mg^2+^ retention provide strong evidence for the emergence and stabilization of the metastable Mg_x_T‐VS_4_ phase rather than a full reversion to the original lattice framework, which deviates from the thermodynamic path and establishes a new equilibrium associated with enhanced Mg storage capability. Figure 5c shows the TEM‐EDS mapping of T‐VS_4_ at states B, C, E, and G. The uniform distribution of V, S, and Ti across all states, demonstrating the well stability of the material at various charged/discharged states. Besides, Mg signals are still detectable even at stage E, corroborating the incomplete Mg^2+^‐extraction observed in XRD and STEM, and pointing toward the stabilization of Mg within a new structural environment. Additional STEM and HRTEM characterizations conducted after 200 cycles (Figure S26) reveal that both the morphology and lattice order of the Mg_x_T‐VS_4_ phase remain well preserved, confirming the structural stability of the metastable framework during extended cycling. Then, we quantitatively assessed the capacity loss by comparing the discharge profiles of the first and second cycles (Figure 5d). The T‐VS_4_ electrode exhibits a capacity loss of 25.31%, nearly half of the 54.16% recorded for pristine VS_4_. This improvement is attributable to the formation of a metastable Mg_x_T‐VS_4_ host during the initial insertion process. The metastable phase prevents the collapse into thermodynamically stable Mg‐rich domains while instead stabilizes a reversible host framework. As a result, active diffusion channels are preserved, suppressing first‐cycle irreversibility. Moreover, the corresponding ex situ XRD in Figure 5e further confirms this structural evolution. The two main peaks at 15.7° and 17.0° correspond to respective the *(110)* and *(020)* planes of monoclinic T‐VS_4_. These two peaks weaken upon discharge due to Mg^2+^ insertion and the formation of a Mg_x_T‐VS_4_ metastable phase, and partially recover during charging from stage C to E. However, even at the fully charged state E, the XRD pattern does not revert to the pristine configuration, again supporting the formation of an electrochemically active metastable phase during Mg^2+^ insertion.

The valence states of V and S in T‐VS_4_ have been systematically evaluated by the high‐resolution XPS, in the Mg^2+^ insertion/extraction process. As shown in Figure 5f, the valence states of Mg are recorded. The Mg 1s peak has not been detected at the pristine state A, however, it is clearly observed in the fully discharged and charged states, further supporting the above analyses that some Mg remain trapped in the structure even at the full‐charged state. In Figure 5g, two peaks at 516.4 eV (V 2p_3/2_ for V^4+^) and 524.1 eV (V 2p_1/2_ for V^4+^) are observed, at the pristine state. Upon discharge, V^4+^ is oxidized to V^5+^, along with the appearance of the new peaks at 517.5 eV (V 2p_3/2_) and 525.1 eV (V 2p_1/2_) [25]. From stage B to C, the V^4+^/V^5+^ ratio decreases from 1.19 to 0.44. During charging, some V^5+^ reverts to V^4+^, resulting in a V^4+^/V^5+^ ratio of 0.99 at state D and 1.86 at state E. In the second discharge cycle, the V^4+^/V^5+^ ratio follows a similar pattern to that observed in the first discharge cycle (0.95 of F and 0.45 of G), excellent electrochemical reversibility of the V element (Figure S27). On the other hand, the valence change of S is present in Figure 5h. For the original T‐VS_4_, the peaks of S_2_^2−^ (162.9 eV at S 2p_3/2_, 164.1 eV at S 2p_1/2_) and S^2−^ (162.3 eV at S 2p_3/2_, 163.4 eV at S 2p_1/2_) are appeared, although the S_2_^2−^ peak possesses a significantly smaller convolution compared to S^2−^. During discharge, the S^2−^ peak intensity increases, while the intensity of S_2_^2−^ decreases. Consequently, the ratio of S_2_^2−^/S^2−^ gradually reduces from 2.56 at state A to 0.48 at state C. Upon charge, the S_2_^2−^/S^2−^ ratio recovers to 0.94 at state D, and 1.77 at state E (Figure S28). It is worth noting that in T‐VS_4_, both V and S participate in the oxidation and reduction reaction during cycling. Ti serves as structural modulator‐introducing sulfur vacancies, expanding the interlayer spacing, and softening the structure, providing pre‐conditions for the metastable Mg_x_T‐VS_4_ formation. It should be noted that this metastable Mg_x_T‐VS_4_ persists throughout the subsequent charge/discharge cycles and underpins the superior electrochemical performance of T‐VS_4_.

## 3 Conclusions

In summary, this work reports, for the first time, the discovery of a metastable Mg_x_T‐VS_4_ phase formed during the initial Mg^2+^ insertion in T‐VS_4_, providing ultra‐sustainable, high capacity, and kinetics‐enhanced MIBs. Significantly enhanced ion diffusion coefficients, high specific capacity of ∼200 mAh g^−1^ at 50 mA g^−1^, excellent rate capability of 64 mAh g^−1^ at 1 A g^−1^, together with outstanding cycling stability with the retention of 80% over 3000 cycles at 1 A g^−1^, have been achieved in the MIBs using such T‐VS_4_. These findings establish a fundamental principle of coupling lattice preconditioning with metastable phase formation to overcome intrinsic kinetic constraints of Mg^2+^ storage, offering a valuable new insight for the design of advanced cathodes in next‐generation ion batteries.

## Funding

This work was supported by China Postdoctoral Science Foundation (No. 2025M770025), National Natural Science Foundation of China (No. 52401291), New Chongqing Youth Innovative Talent Project (No. CSTB2024NSCQ‐QCXMX0018), and Chongqing Postdoctoral Innovation Talents Program. John Wang and team acknowledge the support of Singapore Ministry of Education (MOE) for the support of Tier 2 project (MOE‐T2EP50224‐0020), research conducted at the National University of Singapore.

## Conflicts of Interest

The authors declare no conflicts of interest.

## Supporting information




**Supporting file**: advs73566‐sup‐0001‐SuppMat.docx.

## Data Availability

The data that support the findings of this study are available in the supplementary material of this article.
