# Supplementary material for: Metastable Structure for Ultra‐Sustainable, High Capacity and Kinetics‐Enhanced Magnesium‐Ion Battery
Source: Adv Sci (Weinh). 2026 Jan 4;13(14):e22416. doi: 10.1002/advs.202522416 (PMC12970269; doi:10.1002/advs.202522416)
Supplement: Supplementary file 1 — Supporting file: advs73566‐sup‐0001‐SuppMat.docx. [file ADVS-13-e22416-s001.docx]

Supporting Information

Metastable Structure for Ultra-Sustainable, High Capacity and Kinetics-Enhanced Magnesium-ion Battery

Rongrui Deng, Yumei Wang*, Zhongting Wang*, Xingyang Wang, Chaoneng Dai, Lingxiao Luo, Yue Guo, Jiaqi Peng, Zhenhang Huang, Shuangshuang Tan, Hongyi Li, Fusheng Pan, John Wang*

R. Deng, Y. Wang, X. Wang, Y. Guo, J. Peng, Z. Huang, J. Wang

National University of Singapore (Chongqing) Research Institute

Chongqing, 401123, China

E-mail: yumei_emma@qq.com (Y. Wang), msewangj@nus.edu.sg (J. Wang)

Z. Wang, S. Tan, H. Li, F. Pan

National Engineering Research Center for Magnesium Alloys

College of Materials Science and Engineering, Chongqing University

Chongqing, 400044, China

E-mail: wang.zhongting@cqu.edu.cn (Z. Wang)

X. Wang, J. Wang

Department of Materials Science and Engineering

National University of Singapore

Singapore, 117574, Singapore

C. Dai

School of Materials and Energy

Southwest University

Chongqing 400715, China

L. Luo

College of Materials

Xiamen University

Xiamen, 361005, China

1. **Experimental Section**

**Materials.** Ammonium metavanadate (NH_4_VO_3_), thioacetamide (CH_3_CSNH_2_, TAA), ethylene glycol (EG), sodium metatitanate (Na_2_Ti_3_O_7_), phenylmagnesium chloride (27% in tetrahydrofuran, 2mol/L, PhMgCl/THF), and anhydrous aluminium chloride (AlCl_3_) were purchased from Macklin Co., Ltd. All reagents are analytical reagent without any additional purification.

**Synthesis of T-VS_4_**. 10 mmol of NH_4_VO_3_ was dissolved in 100 mL of deionized water and stirred at 60 °C to obtain a clear solution. To this, 0.0438 g of Na_2_Ti_3_O_7_ was added with continuous stirring. Separately, TAA was dissolved in 100 mL of EG under the same conditions. The two solutions were then combined and stirred for an additional 30 minutes at 70 °C. The mixture was transferred to a Teflon-lined stainless-steel autoclave and heated at 200 °C for 12 hours. The resulting T-VS_4_ precipitate was collected and thoroughly washed with deionized water and absolute ethanol.

**Synthesis of VS_4_**. The pristine VS_4_ was synthesized under identical conditions but without the addition of Na_2_Ti_3_O_7_.

**Preparation of (****PhMgCl)_2_-AlCl_3_ (APC) Electrolyte**: The APC electrolyte was prepared in an argon-filled glove box, where water and oxygen levels were controlled to remain below 0.01 pm, following established protocols. To prepare the electrolyte, 1.0000 g of AlCl_3_ powder was gradually dissolved in 22.5 mL of THF, and stirred for 12 hours. The resulting solution was then slowly added to 7.5 mL of a 2 M PhMgCl/THF solution and stirred for an additional 12 hours to form the APC electrolyte.

**Preconditioning of cathodes**: In order to obtain the metastable T-VS_4_ structure, the cell was initially activated by cycling at a low current density of 50 mA for two cycles before proceeding with subsequent tests.

**Electrochemical Tests**. The working electrode was prepared by mixing T-VS_4_/ VS_4_ cathodes, conductive carbon black (Super P), and polyvinylidene difluoride (PVDF) in an 8:1:1 mass ratio. The resulting slurry was coated onto carbon paper and dried in a vacuum oven at 60 °C for 24 hours before being cut into small pieces. Polished high-purity magnesium foil (>99.9%, 0.1 mm, Scimaterials) served as the anode. Coin-type CR2032 cells were assembled in an argon-filled glove box (Mikrouna) with water and oxygen concentrations maintained below 0.1 ppm, following established protocols. Glass fibers separated the cathode and anode. To prevent electrochemical corrosion of the electrolyte and the stainless-steel cell case under high voltage, the battery case underwent a special anti-corrosion treatment. The galvanostatic charge/discharge performance, with a voltage range of 0.3 V to 2.1 V (*vs.* Mg^2+^/Mg). Galvanostatic intermittent titration (GITT) were tested using a battery test system (Neware), cycled at a current density of 50 mA g^−1^ for 5 minutes, followed by a 5-minute open-circuit relaxation to determine the equilibrium potential. Cyclic voltammetry (CV) was conducted on an electrochemical workstation (CHI 660E), and electrochemical impedance spectroscopy (EIS) was performed using the Autolab-302N and PARSTAT 4000 electrochemical workstation.

**Characterization and Testing**: The phase composition of the samples was determined by X-ray Diffraction (Shimadzu-6100, Cu Kα radiation) with a 2θ angle range from 10° to 90° and a step size of 0.05°. The multi-element chemical composition was analyzed by X-ray Photoelectron Spectroscopy (Shimadzu-1800). Scanning Transmission Electron Microscopy (Talos-F200S) was used to investigate the material morphology. To further study the chemical structure of the materials Fourier Transform Infrared spectroscopy (Nicolet-iS50) was utilized.

**Computational methods**: Models of the VS_4_ bulk structure and T-VS_4_ were constructed using density functional theory (DFT) within the projector augmented plane-wave method, as implemented in the Vienna Ab-initio Simulation Package (VASP). The DFT-D3 correction was applied to account for weak interactions between atoms. A plane wave cut-off energy of 450 eV and an energy convergence criterion of 10^−5^ eV were used in solving the Kohn-Sham equations iteratively. The the Electron Localization Function (ELF) for VS_4_ was obtained by slicing along V atoms, with a saturation level from 0 to 1 e/Å^−3^. The climbing image nudged elastic band (CI-NEB) method was employed to determine the migration paths of Mg^2+^, with an energy convergence criterion of 10^−7^ eV for the search of the transition state.

1. **Equation**

$D=\frac{4}{\pi\tau}(\frac{m_{A}V_{M}}{M_{A}S})^{2}(\frac{\triangle E_{s}}{\triangle E_{t}})^{2}$ (Equation S1)

Where *τ* is the resting time (s), *m_A_* is the mass of the active material (g); *V_M_* is the molar volume of the active material (cm^3^ mol^−1^); *M_A_* is the molar mass of the material (g mol^−1^); *S* is the effective surface area of the electrode (cm^2^); *ΔE_s_* is the voltage change during charge/discharge (V); *ΔE_t_* is the voltage change from resting to equilibrium (V).

The internal resistance, calculated from the GITT curves (**Equation S1**), provides a crucial assessment of Mg^2+^-diffusion properties in T-VS_4_/VS_4_ electrodes.

 (Equation S2)

Where *V_QOCV_* is the quasi-open circuit voltage (V); *V_CCV_* is the closed-circuit voltage (V); *I* is the applied current (A). As shown in **Figure S2**.

The average internal resistance for T-VS_4_ is 1.42 Ω and 2.33 Ω during discharge and charge, both much lower than 7.59 Ω (discharge) and 11.28 Ω (charge) for VS_4_.

$i=av^{b}$ (Equation S3)

$\frac{i}{v^{1/2}}=k_{1}v^{1/2}+k_{2}\text{ }$ (Equation S4)

When *b* is closing to 1, the storage mechanism is predominantly surface-capacitive, while when it reaching 0.5, a diffusion-controlled process wins.

The DRT transformation can be implemented using the following Equation^[1]^:

$Z(f)=R_{0}+i2\pi fL_{0}+Z_{pol}(f)$ (Equation S5)

$Z_{pol}(f)=\int_{0}^{+\infty} \frac{\gamma(\tau)}{1+i2\pi f\tau}d\tau$ (Equation S6)

where *f* denotes the frequency; *R*_0_​ is the ohmic resistance; *i* is the imaginary unit; *L*_0_​ indicates the inductance; *τ* is the characteristic time constant, and *γ*(*τ*) is the distribution of time constants across various electrochemical processes. The distribution function of the relaxation time, *γ*(*τ*), is described by^[2]^:

$\int_{0}^{\infty} \gamma(\tau)d\tau=1$ (Equation S7)

1. **Table**

**Table S1**. The bond length and bond angle of the VS_4_, T-VS_4_, and Mg_x_T-VS_4_

|  | bond length of V-S (Å) | bond length of Ti-S (Å) | bond angle of S-V-S (°) | bond angle of S-Ti-S (°) |
| --- | --- | --- | --- | --- |
| VS_4_ | 2.37 | / | 88.23 | / |
| T-VS_4_ | 2.38 | 2.58 | 86.48 | 92.12 |
| Mg_x_T-VS_4_ | 2.42 | 2.62 | 82.94 | 89.18 |

**Table S2**. Comparison of T-VS_4_ cathode with other reported VS_4_-based cathodes for magnesium-ion batteries.

| Cathode | Electrolyte | Anode | Voltage range  (V *vs.* Mg^2+^/Mg) | Coulombic efficiency | Discharge specific capacity  (mAh g^–1^) | Reference |
| --- | --- | --- | --- | --- | --- | --- |
| This work | APC | Mg | 0.3~2.1 | > 99 % | 205.4 mAh g^−1^ at 50 mA g^−1^, over 3000 cycles | / |
| Mo-VS_4_/N-TG | APC | Mg | 0.2~2.1 | > 96 % | 140 mAh g^−1^ at 50 mA g^−1^, 1200 cycles under 500 mA g^−1^ | ^[3]^ |
| PVP-VS_4_ | APC | Mg | 0.2~2.1 | > 99 % | 140 mAh g^−1^ at 50 mA g^−1^ | ^[4]^ |
| Mo-VS_4_ | APC | Mg | 0.2~2.1 | > 99 % | 120 mAh g^−1^ at 50 mA g^−1^ over 350 cycles, about 70 mAh g^−1^  at 500 mA g^−1^ | ^[5]^ |
| Atomic VS_4_ Chain | APC | Mg | 0.3~2.2 | ~100 % | ~160 mAh g^−1^ at 100 mA g^−1^ and  74 mAh g^−1^ at 500 mA g^−1^ after 800 cycles | ^[6]^ |
| F-VS_4_ | APC | Mg | 0.2~2.2 | > 90 % | 80 mAh g^−1^ at 50 mA g^−1^ | ^[7]^ |
| CNT@VS_4_ | APC | Mg | 0.3~2.2 | ~100 % | 170 mAh g^−1^ at 100 mA g^−1^, 76.3 mAh g^−1^ after 800 cycles at 500 mA g^−1^ | ^[8]^ |

1. **Figures**


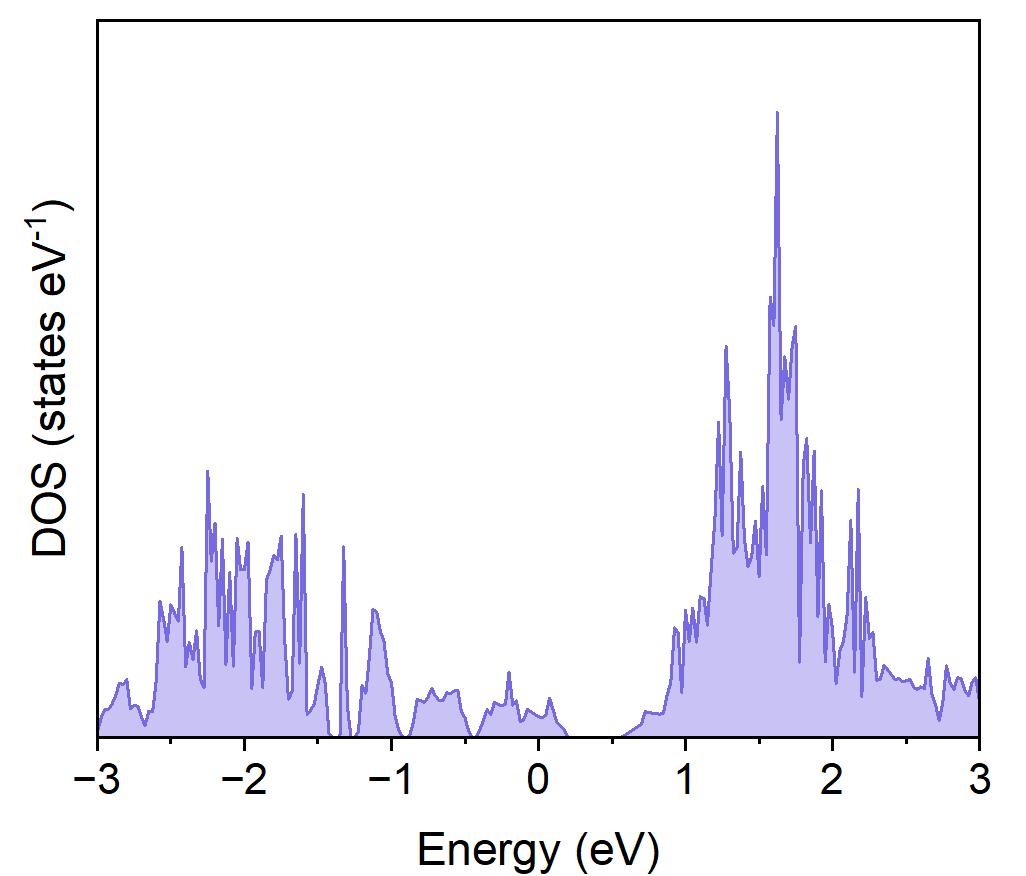


**Figure S1.** Calculated density of states of VS_4_.


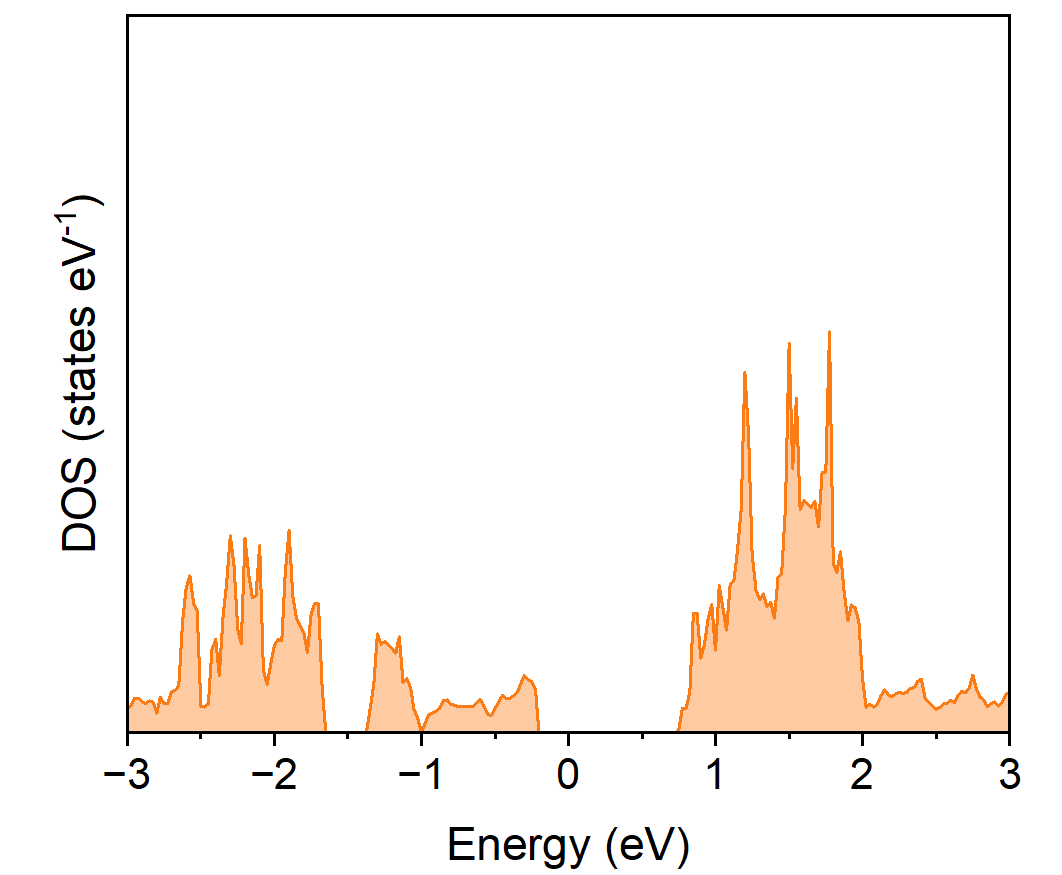


**Figure S2.** Calculated density of states of T-VS_4_.


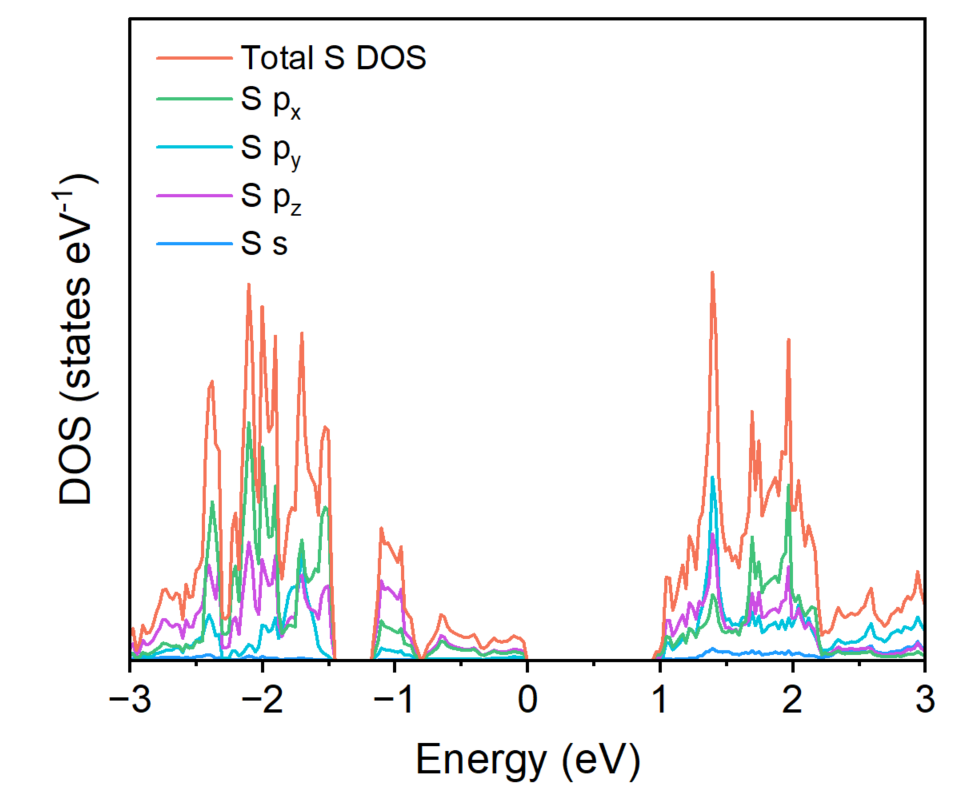


**Figure S3.** Calculated density of states of S in VS_4_.


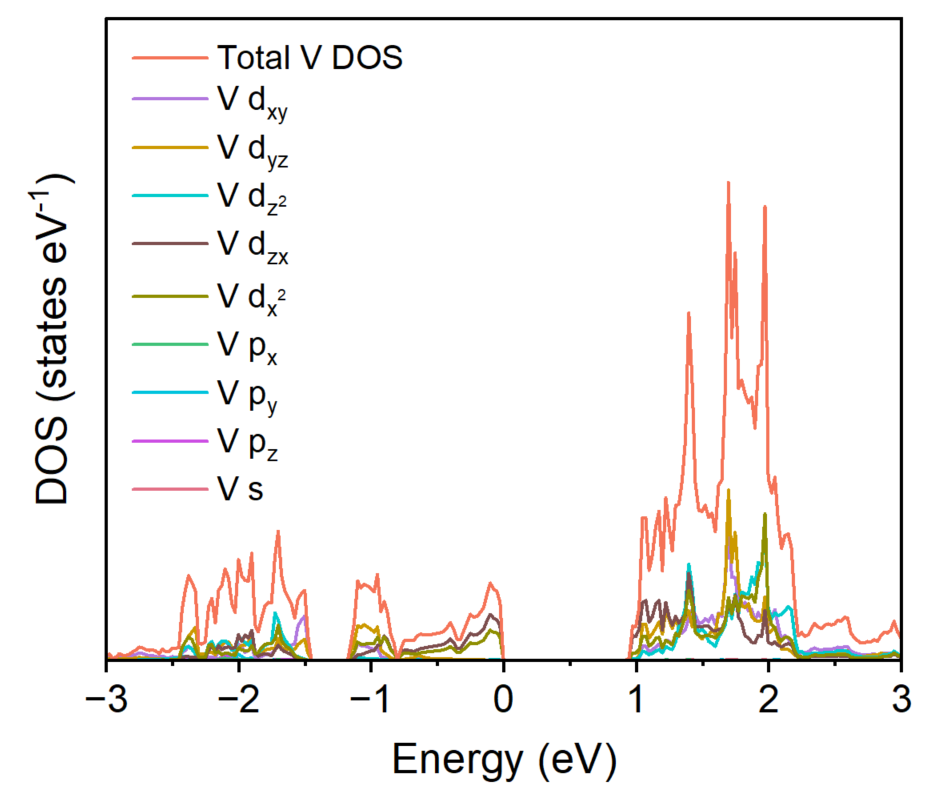


**Figure S4.** Calculated density of states of V in VS_4_.


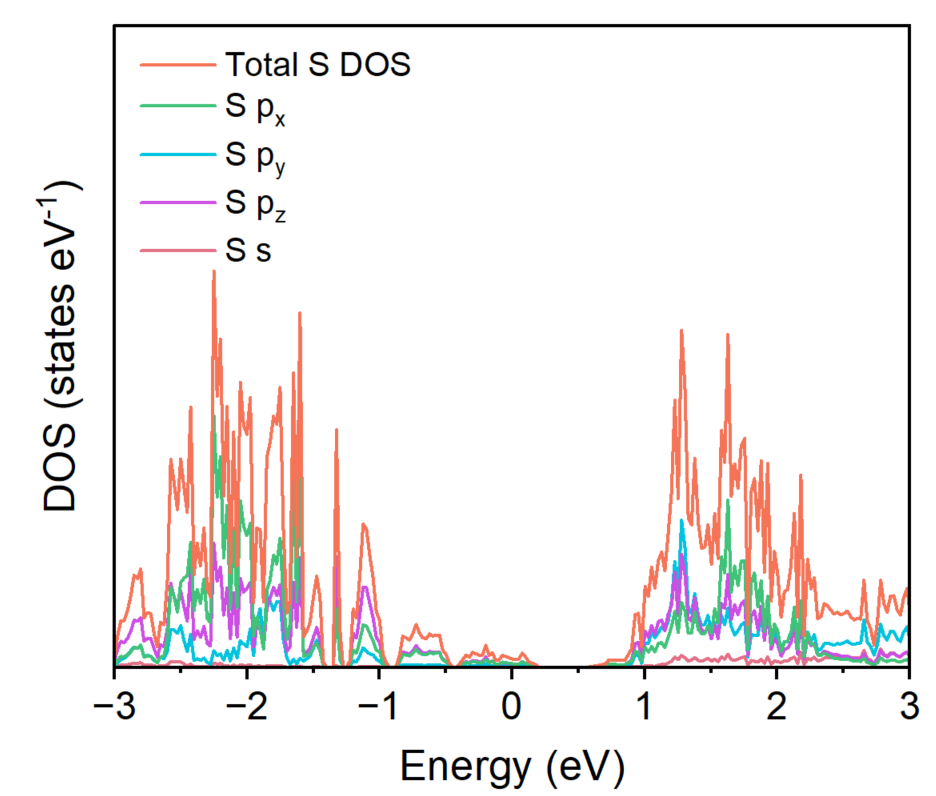


**Figure S5.** Calculated density of states of S in T-VS_4_.


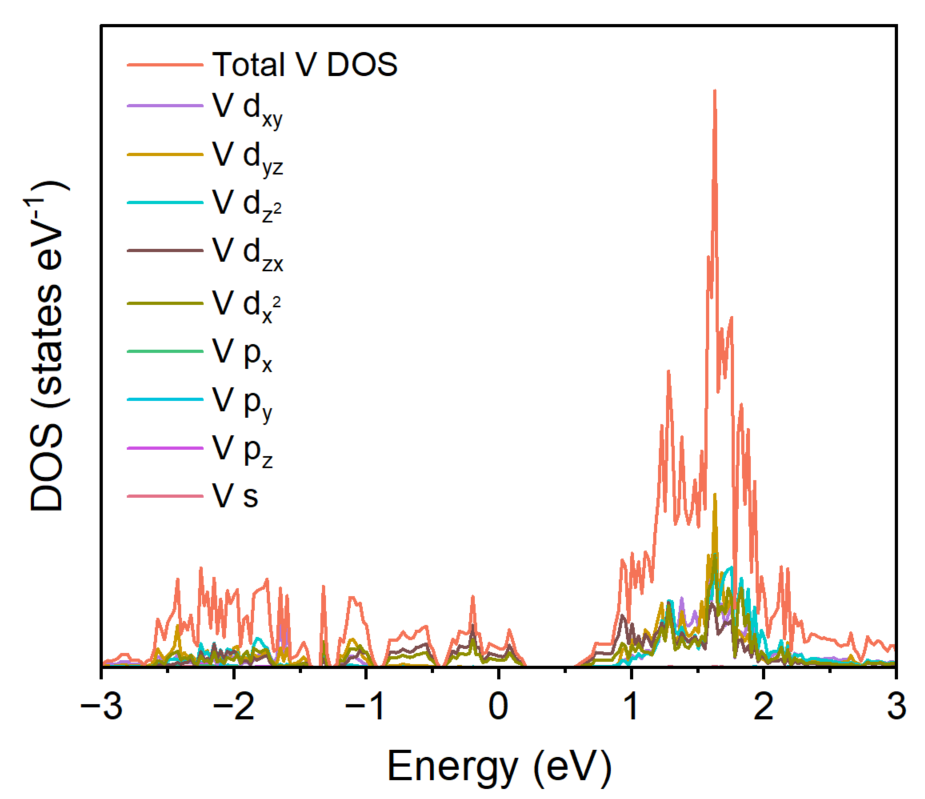


**Figure S6.** Calculated density of states of V in T-VS_4_.


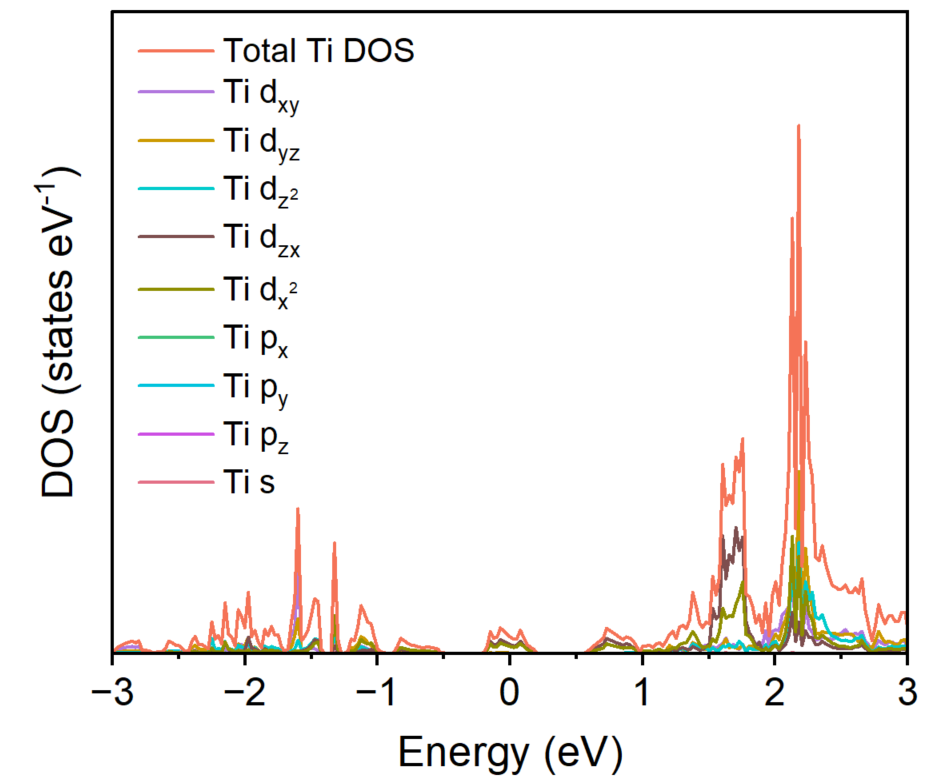


**Figure S7.** Calculated density of states of Ti in T-VS_4_.


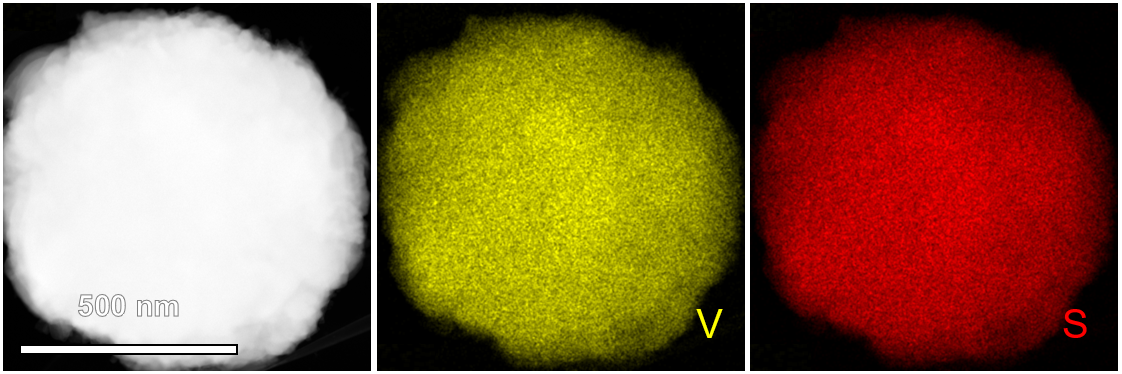


**Figure S8.** HAADF-TEM and corresponding EDS mapping images of VS_4_.


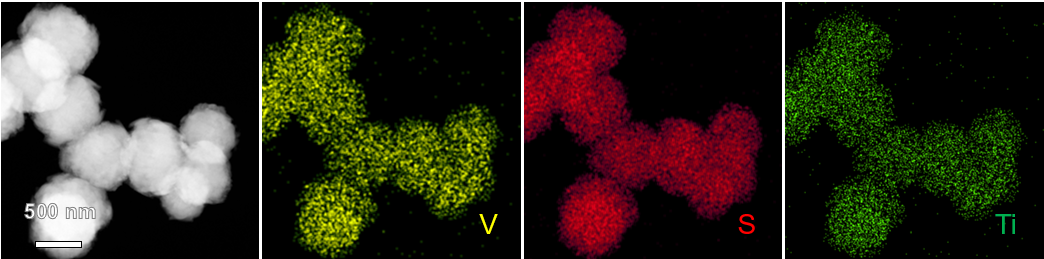


**Figure S9.** HAADF-TEM and corresponding EDS mapping images of T-VS_4_.


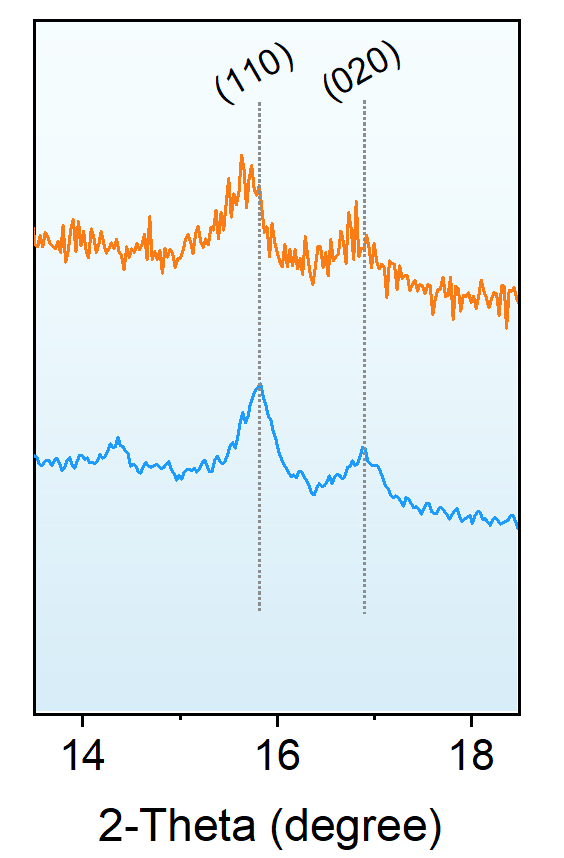


**Figure S10.** Local enlarged XRD results of the T-VS_4_ and VS_4_


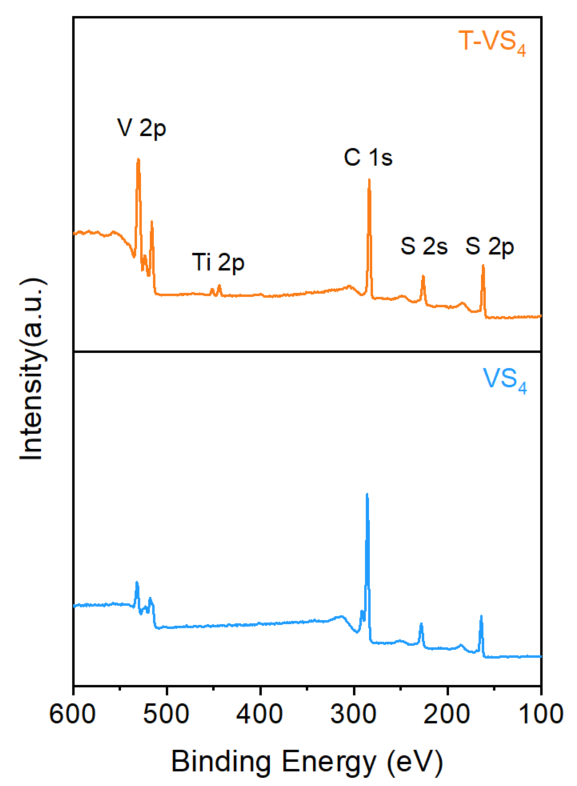


**Figure S11.** Survey XPS spectra of the T-VS_4_ and VS_4_


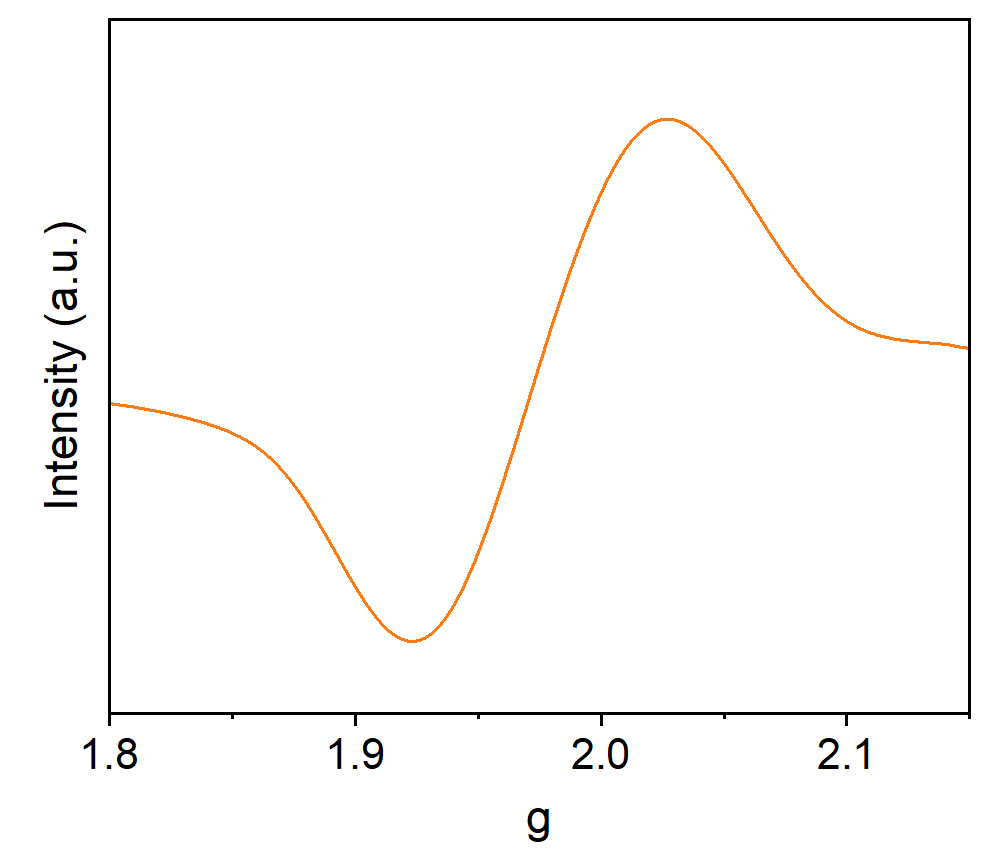


**Figure S12.** EPR spectroscopy of T-VS_4_.


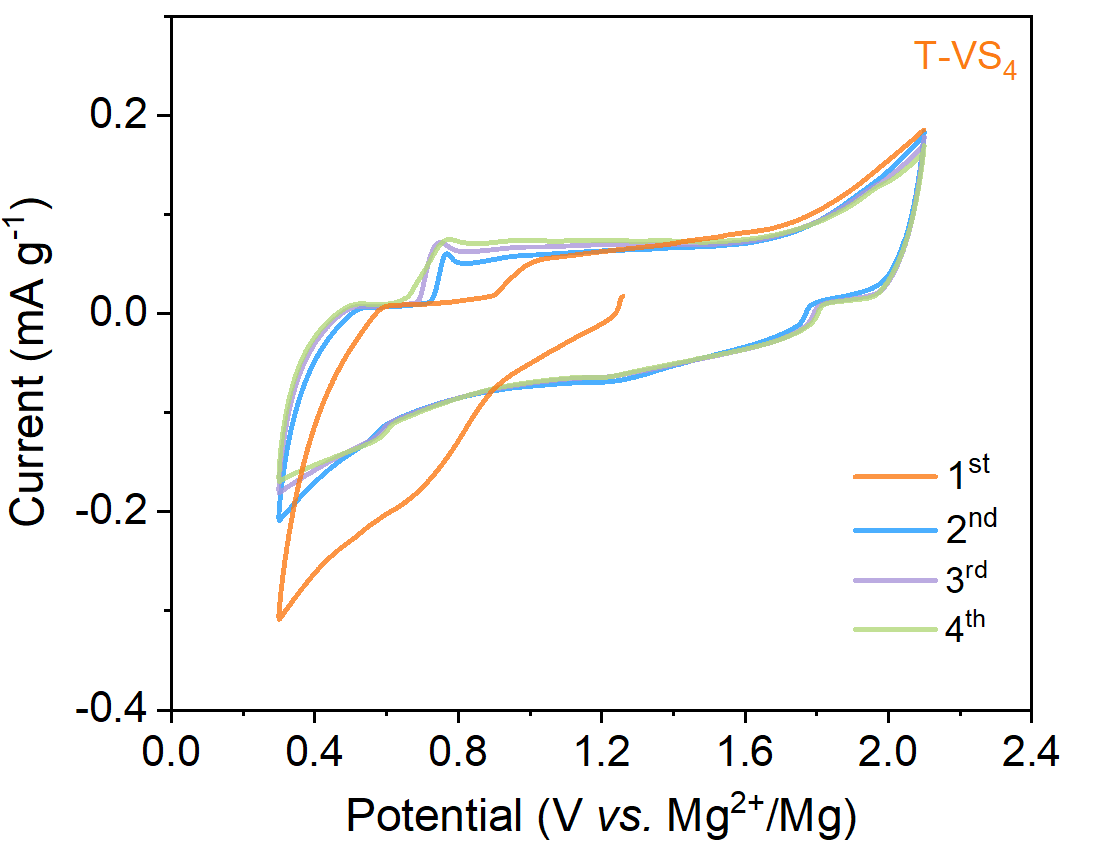


**Figure S13.** CV curves of the T-VS_4_ cathode.


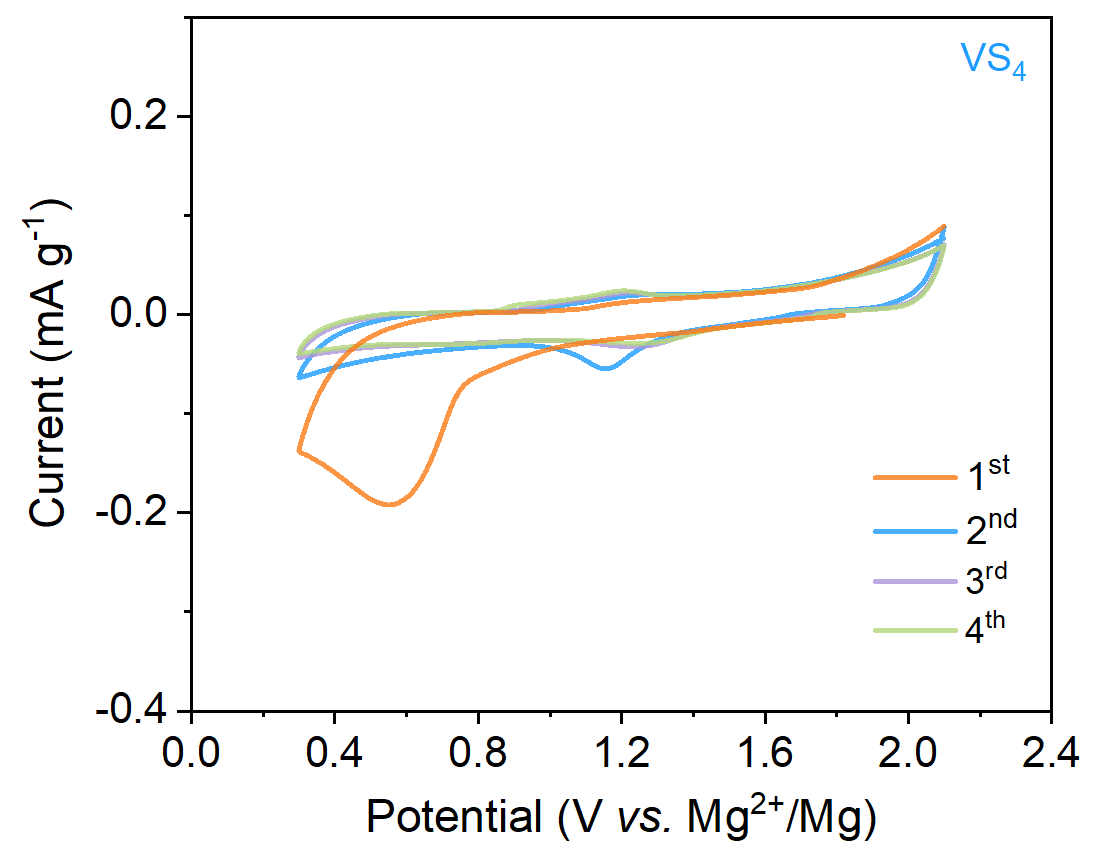


**Figure S14.** CV curves of the VS_4_ cathode.


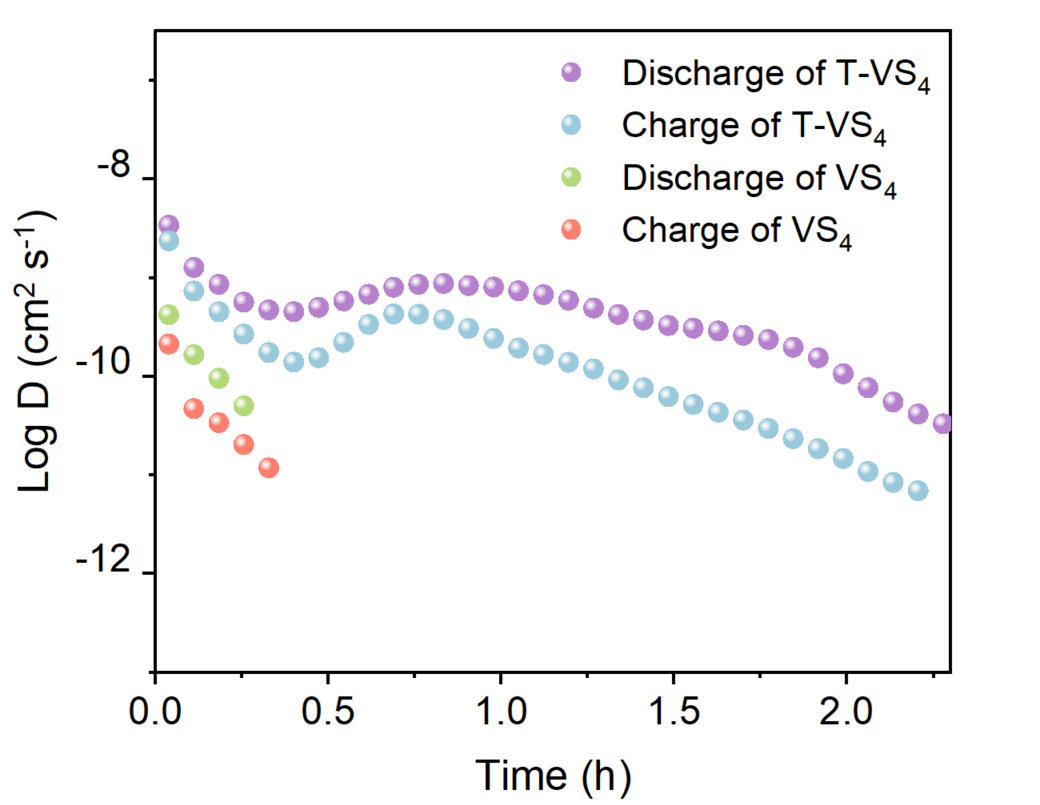


**Figure S15.** Mg^2+^ diffusion coefficient of T-VS_4_ and VS_4_ cathodes.


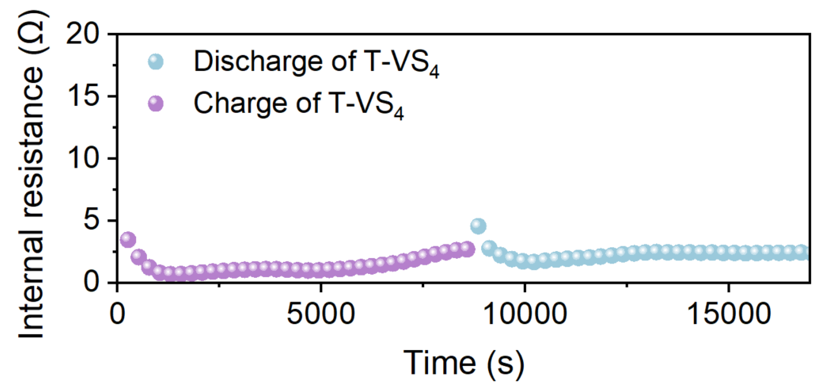


**Figure S16.** The calculated internal resistance based on GITT of T-VS_4_ cathode.


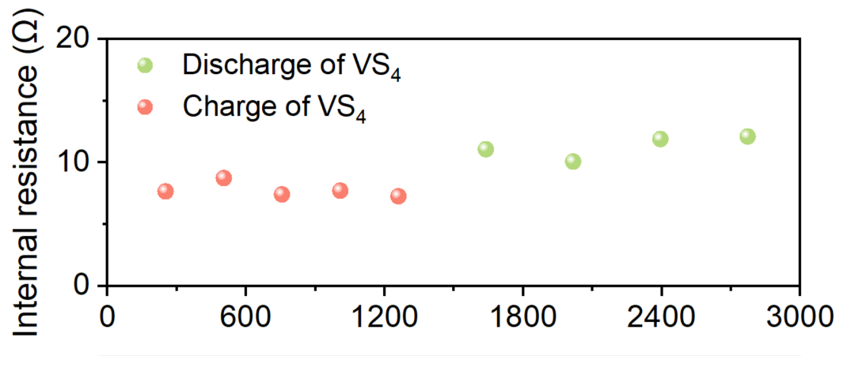


**Figure S17.** Calculated internal resistance based on GITT of VS_4_ cathode.


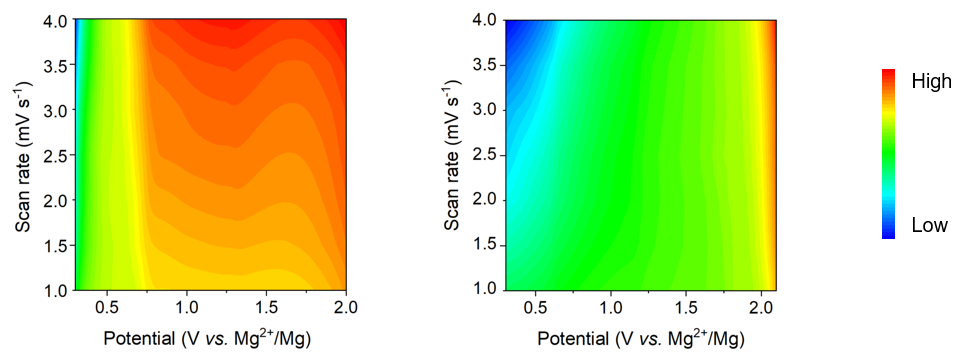


**Figure S18.** CV curves of T-VS_4_ at various scan rates from 1.0 to 4.0 mV s^–1^.

The shapes of the CV peaks for both VS_4_ and T-VS_4_ remain consistent with increasing scan rates, with only the peak currents rising, indicating effective current adaptation. The relationship between the measured current (*i*) and the scan rate (*v*) is described by **Equation S3**, which distinguishes the surface capacity from the diffusion-controlled capacity. The *b* value is an empirical parameter and ranging between 0.5 and 1, is determined by linearly fitting log (*i*) *v.s*. log (*v*), based on the contour plots of the CV curves shown in **Figure S18-19** and following **Equation S4**. As illustrated in **Figure S20**, the *b* values for the three peaks of T-VS_4_ are 0.6314, 0.6624, and 0.5294, with R^2^ values exceeding 0.99. These results indicate a predominant diffusion controlled ion storage mechanism in T-VS_4_. In contrast, VS_4_ cathode exhibits *b* values of 0.6373, 0.8482, and 0.813 (**Figure S21**), suggesting that its capacity is predominantly controlled by surface pseudo-capacitance.


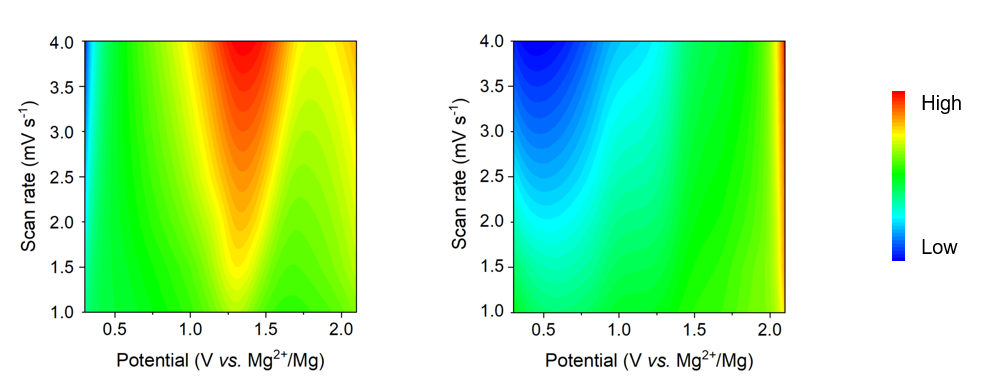


**Figure S19.** CV curves of VS_4_ at various scan rates from 1.0 to 4.0 mV s^–1^.


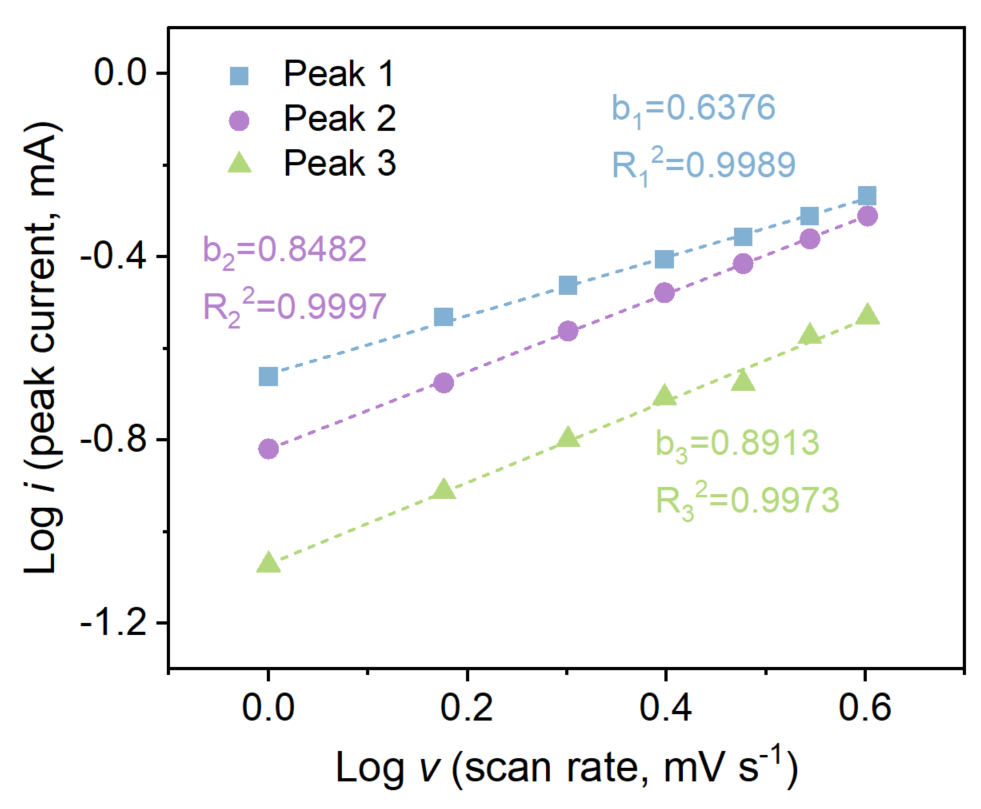


**Figure S20.** The fitted lines for determining *b* values and corresponding fitting degree *R^2^* of VS_4_.


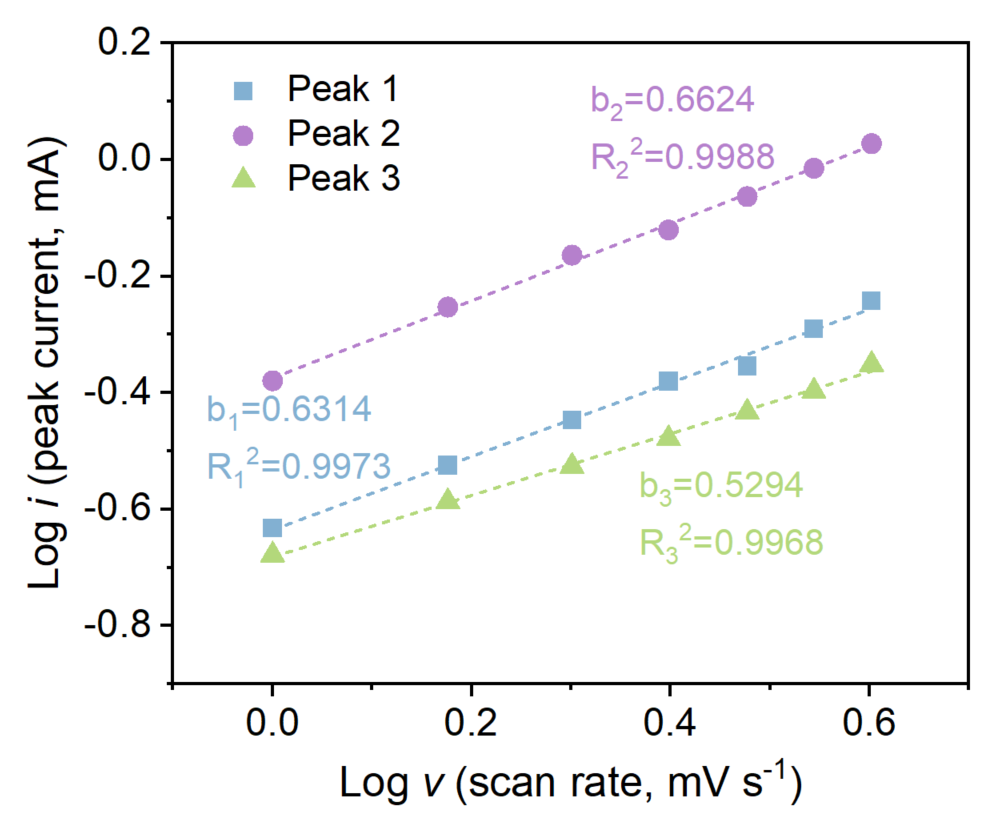


**Figure S21.** The fitted lines for determining *b* values and corresponding fitting degree *R^2^* of T-VS_4_.


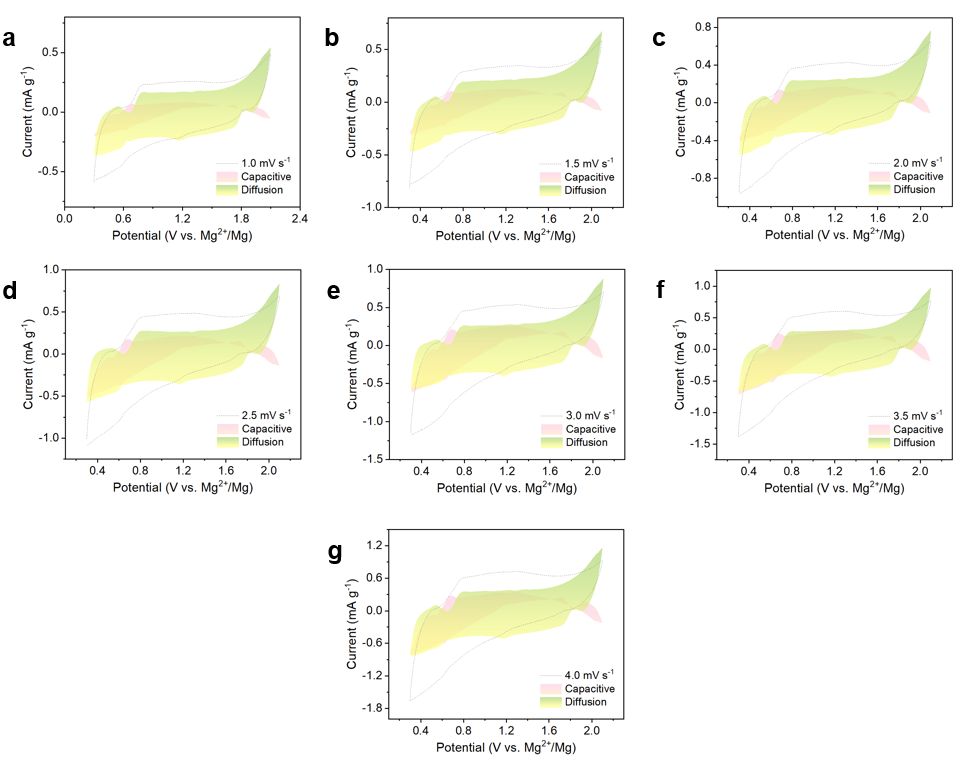


**Figure S22.** Pseudocapacitive and diffusion contribution of T-VS_4_ cathode with scan rate from 1.0 to 4.0 mV s^–1^. a) of 1.0 mV s^–1^; b) of 1.5 mV s^–1^; c) of 2.0 mV s^–1^; d) of 2.5 mV s^–1^; e) of 3.0 mV s^–1^; f) of 3.5 mV s^–1^; g) of 4.0 mV s^–1^.

The contributions of capacitive behavior and diffusion-controlled effects are illustrated in **Figure S22-23**. At a scan rate of 1.0 mV s^−1^, the diffusion-controlled capacity is 84.5%, and it decreases to 72.6% when the scan rate increases to 4.0 mV s^−1^. The dominance of diffusion-controlled behavior in T-VS_4_ indicates that the formation of the metastable Mg_x_T-VS_4_ phase upon initial Mg^2+^ intercalation plays a crucial role in enhancing ion transport. This metastable phase, with its dynamic structural flexibility, provides energetically favorable pathways for Mg^2+^ migration, thereby improving ion storage and enhancing electrochemical performance. In contrast, the VS_4_ electrode consistently exhibits a dominant pseudocapacitive effect (**Figure S24-25**), which hampers efficient Mg^2+^ migration and limits overall performance. The results from the capacity ratio mechanism align with theoretical analysis, simulation calculations, and electrochemical data, reinforcing the superior kinetics and performance of T-VS_4_, driven by the unique metastable phase.


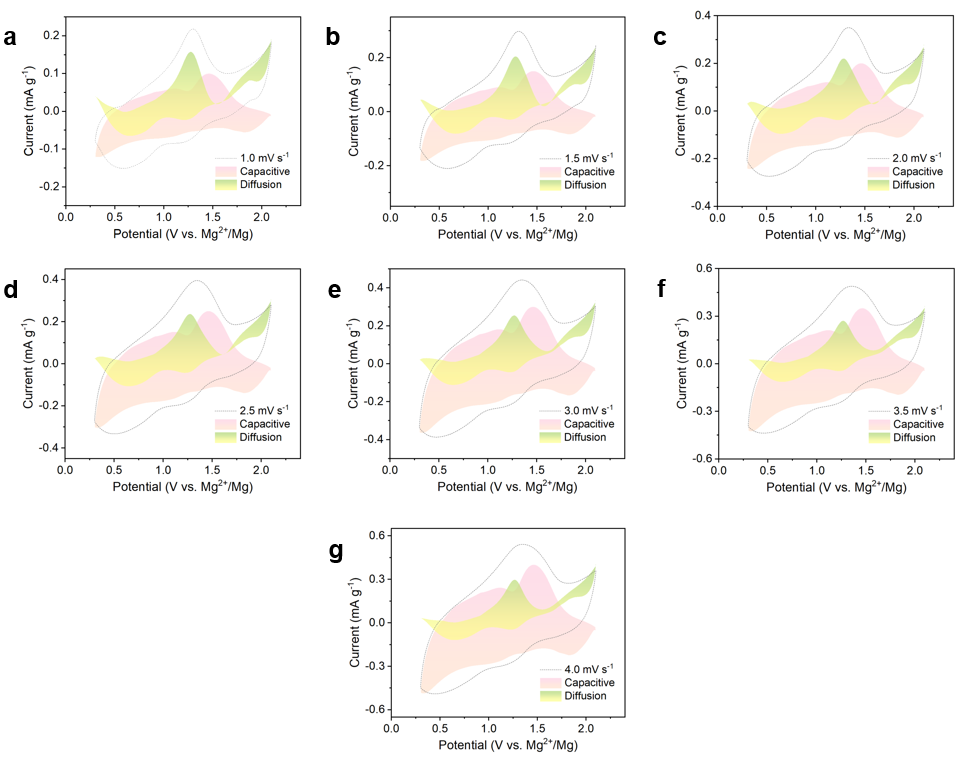


**Figure S23.** Pseudocapacitive and diffusion contribution of VS_4_ cathode with scan rate from 1.0 to 4.0 mV s^–1^. a) of 1.0 mV s^–1^; b) of 1.5 mV s^–1^; c) of 2.0 mV s^–1^; d) of 2.5 mV s^–1^; e) of 3.0 mV s^–1^; f) of 3.5 mV s^–1^; g) of 4.0 mV s^–1^.

**
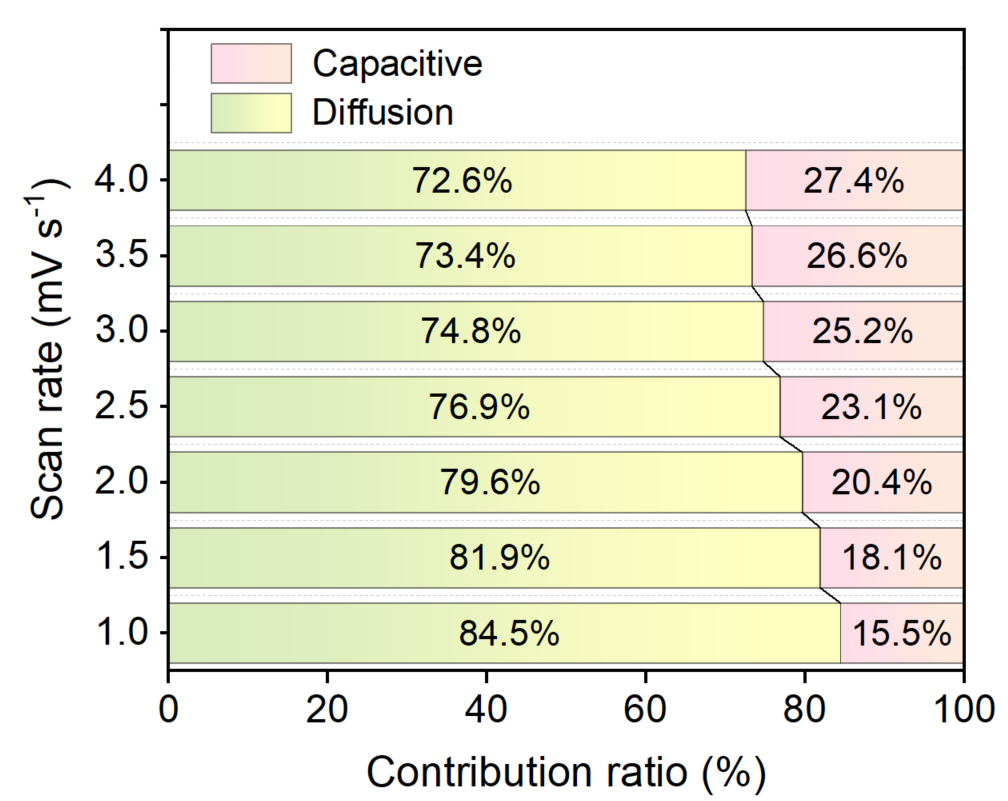
**

**Figure S24.** The capacitive contribution ratio of T-VS_4_.


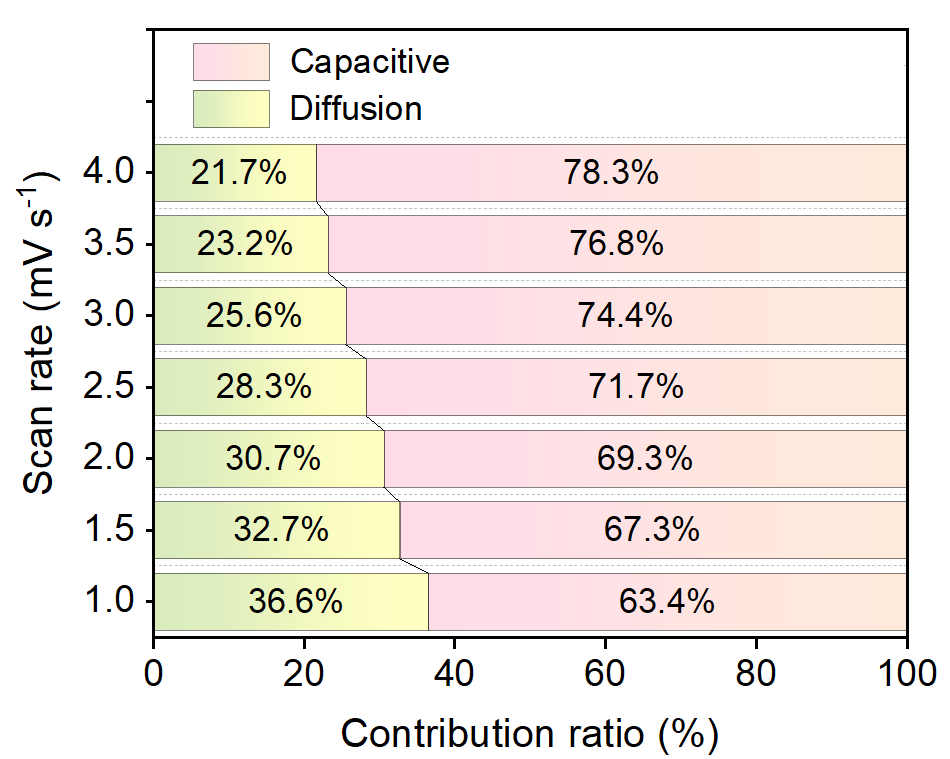


**Figure S25.** The capacitive contribution ratio of VS_4_.


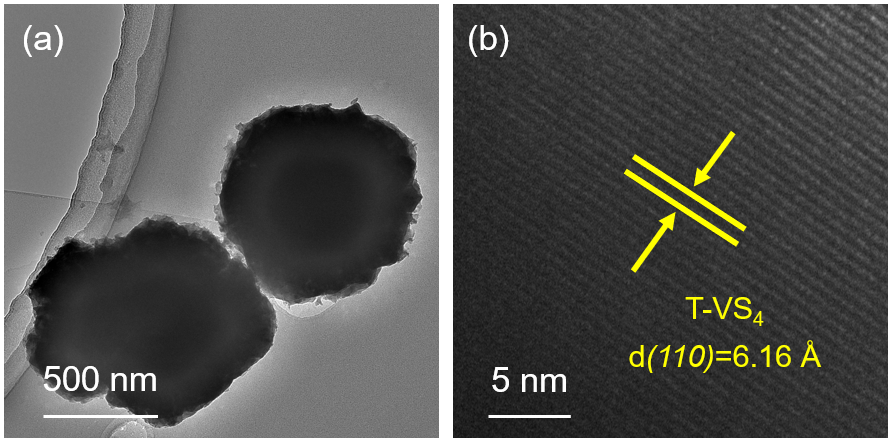


**Figure S26.** (a) STEM and (b) HRTEM images of T-VS_4_ cathode after 200 cycles.


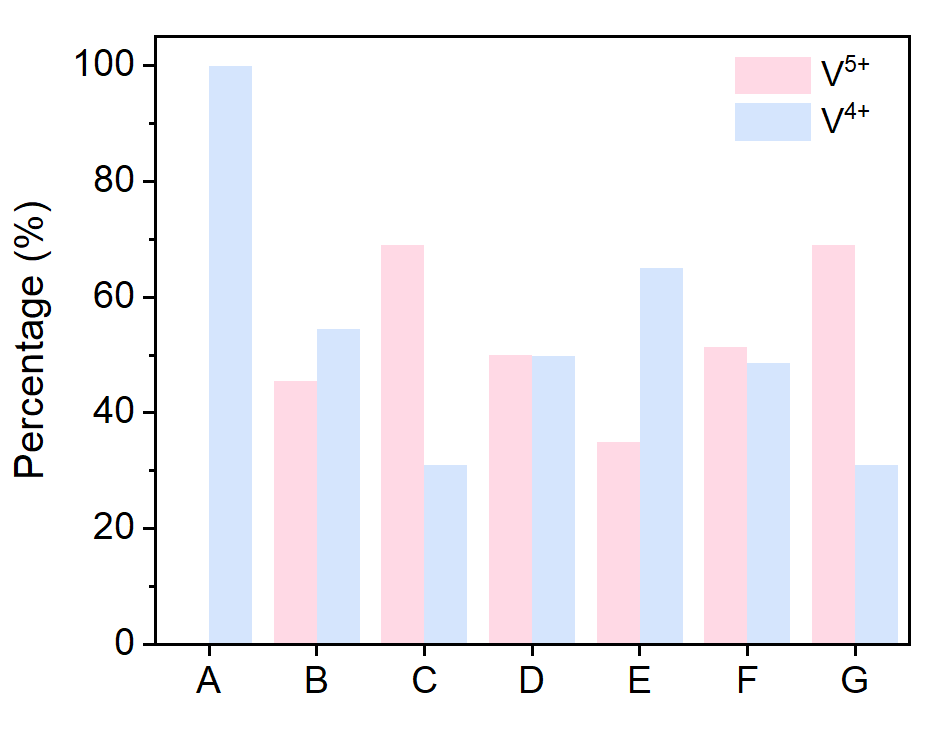


**Figure S27.** The percentage of V^4+^ and V^5+^ at typical stages of electrochemical cycling.


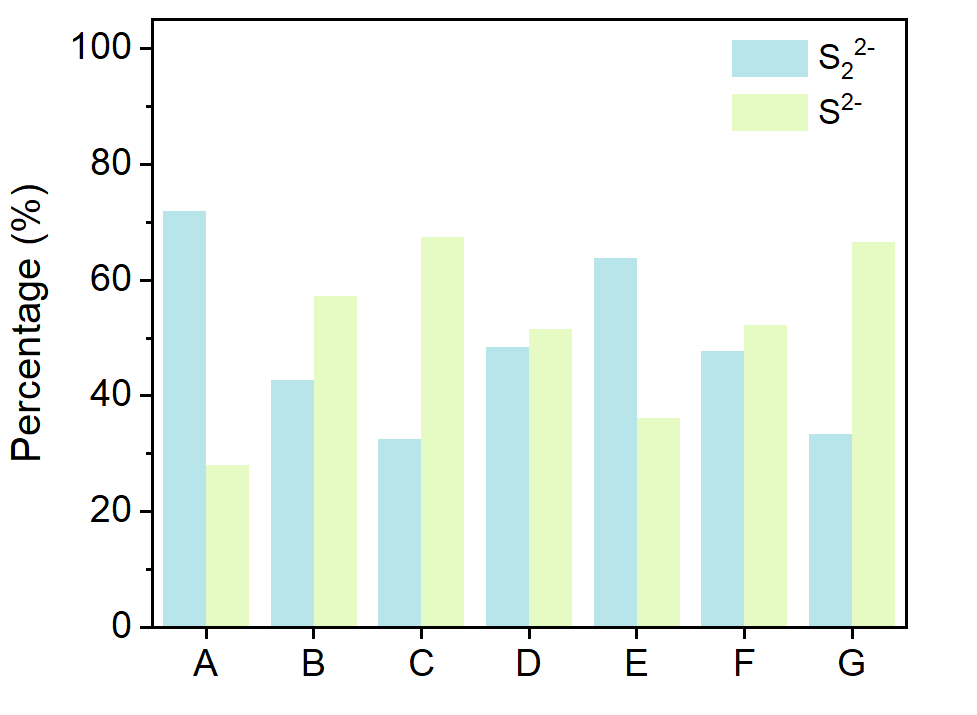


**Figure S28.** The percentage of S_2_^2–^ and S_2_^–^ at typical stages of electrochemical cycling.

**Reference**

[1] a) J. Huang, N. P. Sullivan, A. Zakutayev, R. O’Hayre, *Electrochimica Acta* **2023**, 443; b) L. Zhang, X. Hong, W. Xu, D. Ruan, *Journal of Energy Storage* **2024**, 94.

[2] a) Y. Lu, C.-Z. Zhao, J.-Q. Huang, Q. Zhang, *Joule* **2022**, 6, 1172; b) Y. Lu, C.-Z. Zhao, J.-K. Hu, S. Sun, H. Yuan, Z.-H. Fu, X. Chen, J.-Q. Huang, M. Ouyang, Q. Zhang, *Science Advances* **2022**, 8, eadd0510.

[1] a) J. Huang, N. P. Sullivan, A. Zakutayev, R. O’Hayre, *Electrochimica Acta* **2023**, 443; b) L. Zhang, X. Hong, W. Xu, D. Ruan, *Journal of Energy Storage* **2024**, 94.

[2] a) Y. Lu, C.-Z. Zhao, J.-Q. Huang, Q. Zhang, *Joule* **2022**, 6, 1172; b) Y. Lu, C.-Z. Zhao, J.-K. Hu, S. Sun, H. Yuan, Z.-H. Fu, X. Chen, J.-Q. Huang, M. Ouyang, Q. Zhang, *Science Advances* **2022**, 8, eadd0510.

[3] S. Ding, X. Dai, Y. Tian, G. Song, Z. Li, A. Meng, L. Wang, G. Li, W. Wang, J. Huang, S. Li, *ACS Appl Mater Interfaces* **2021**, 13, 54005.

[4] S. Q. Ding, X. Dai, Z. J. Li, C. S. Wang, A. Meng, L. Wang, G. C. Li, J. F. Huang, S. X. Li, *Energy Storage Materials* **2022**, 47, 211.

[5] Y. Wang, Z. Liu, C. Wang, X. Yi, R. Chen, L. Ma, Y. Hu, G. Zhu, T. Chen, Z. Tie, J. Ma, J. Liu, Z. Jin, *Adv Mater* **2018**, 30, e1802563.

[6] S. Q. Ding, Z. J. Li, X. Dai, C. L. Sun, A. L. Meng, *Chemical Engineering Journal* **2021**, 417.

[7] Z. J. Li, S. Q. Ding, J. F. Yin, M. Zhang, C. L. Sun, A. Meng, *Journal of Power Sources* **2020**, 451.

[8] J. Li, Y. Xu, Y. He, Z. Zhang, C. Zhu, X. Zhou, *J Phys Chem Lett* **2022**, 13, 5726.
